# Mechanisms That Determine the Internal Environment of the Developing Brain: A Transcriptomic, Functional and Ultrastructural Approach

**DOI:** 10.1371/journal.pone.0065629

**Published:** 2013-07-02

**Authors:** Shane A. Liddelow, Katarzyna M. Dziegielewska, C. Joakim Ek, Mark D. Habgood, Hannelore Bauer, Hans-Christian Bauer, Helen Lindsay, Matthew J. Wakefield, Nathalie Strazielle, Ingrid Kratzer, Kjeld Møllgård, Jean-François Ghersi-Egea, Norman R. Saunders

**Affiliations:** 1 Department of Pharmacology, the University of Melbourne, Victoria, Australia; 2 Department of Medical Biology, the University of Melbourne, Victoria, Australia; 3 Department of Genetics, the University of Melbourne, Victoria, Australia; 4 Department of Neurobiology, Stanford University, Stanford, California, United States of America; 5 Institute of Neuroscience and Physiology, University of Gothenburg, Gothenburg, Sweden; 6 Department of Organismic Biology, University of Salzburg, Salzburg, Austria; 7 Paracelsus Medical University, Salzburg, Austria; 8 Walter and Eliza Hall Institute, Melbourne, Victoria, Australia; 9 Lyon Neuroscience Research Center, INSERM, U1028, Lyon, France; 10 Brain-i, Lyon, France; 11 Institute of Cellular and Molecular Medicine, University of Copenhagen, Copenhagen, Denmark; Biological Research Centre of the Hungarian Academy of Sciences, Hungary

## Abstract

We provide comprehensive identification of embryonic (E15) and adult rat lateral ventricular choroid plexus transcriptome, with focus on junction-associated proteins, ionic influx transporters and channels. Additionally, these data are related to new structural and previously published permeability studies. Results reveal that most genes associated with intercellular junctions are expressed at similar levels at both ages. In total, 32 molecules known to be associated with brain barrier interfaces were identified. Nine claudins showed unaltered expression, while two claudins (6 and 8) were expressed at higher levels in the embryo. Expression levels for most cytoplasmic/regulatory adaptors (10 of 12) were similar at the two ages. A few junctional genes displayed lower expression in embryos, including 5 claudins, occludin and one junctional adhesion molecule. Three gap junction genes were enriched in the embryo. The functional effectiveness of these junctions was assessed using blood-delivered water-soluble tracers at both the light and electron microscopic level: embryo and adult junctions halted movement of both 286Da and 3kDa molecules into the cerebrospinal fluid (CSF). The molecular identities of many ion channel and transporter genes previously reported as important for CSF formation and secretion in the adult were demonstrated in the embryonic choroid plexus (and validated with immunohistochemistry of protein products), but with some major age-related differences in expression. In addition, a large number of previously unidentified ion channel and transporter genes were identified for the first time in plexus epithelium. These results, in addition to data obtained from electron microscopical and physiological permeability experiments in immature brains, indicate that exchange between blood and CSF is mainly transcellular, as well-formed tight junctions restrict movement of small water-soluble molecules from early in development. These data strongly indicate the brain develops within a well-protected internal environment and the exchange between the blood, brain and CSF is transcellular and not through incomplete barriers.

## Introduction

Understanding the role of brain barrier mechanisms in normal brain development and possible deleterious effects should these mechanisms be dysfunctional is important from a clinical perspective. The understanding of whether or not drugs/toxins may have access to the vulnerable developing brain is critical, regardless of whether this movement is via passive paracellular routes or the functioning of transcellular exchange mechanisms across barrier interfaces. Control of influx and efflux exchange mechanisms together with intercellular junction-associated proteins – generally referred to as brain barrier mechanisms – provide the basis for the well-known stability and composition of the internal environment of the adult brain. In the developing brain however, the status of this stability has been a matter of some dispute, with many believing that the brain barriers are absent, leaky or immature [1]–[4] – as has been extensively reviewed [5], [6]. The developing brain is necessarily immature compared to that of the adult, but the real focus should be on the functional status of the barrier mechanisms in embryos, fetuses and infants, compared to adults. In particular, as we have proposed previously, exchange mechanisms across the blood-cerebrospinal fluid (CSF) barrier at the level of the choroid plexuses within the cerebral ventricles is of special importance in early development, at a time when the brain is poorly vascularised [7], [8] and the choroid plexuses are already well developed [9], [10]. Protective barriers of the brain are dependent on junctional complexes at the interfaces between blood and the central nervous system, including epithelial cells of the choroid plexuses (blood-cerebrospinal fluid barrier) and endothelial cells of brain capillaries (blood-brain barrier). It is accepted that in the adult plexus continuous functional tight junctions between adjacent epithelial cells control access of molecules to the CSF and thence to the brain [11]. The molecular composition and complexity of these junctions in the developing brain in relation to their “tightness” (i.e. permeability properties) have been controversial [5], [6], [12]. Here we present a transcriptome analysis of embryonic and adult rat choroid plexus, combining RNA sequencing datasets with physiological experiments that demonstrate no paracellular movement of small water-soluble molecules through these junctional complexes. It has been reported recently, based on microarray studies in embryonic mouse choroid plexus [13] that many intercellular junction-related and ion transporter and ion channel genes are already expressed in embryonic choroid plexus, including many that are expressed at a higher level or even uniquely in the embryonic choroid plexus. A qRT-PCR study of the tight junction claudin genes in developing rat choroid plexus has also shown early expression and age-related change in expression of this gene family [14].

The present study confirms and extends these findings in the embryonic and adult rat choroid plexus and includes a comprehensive analysis of ion transporters and channel genes expression. These molecular studies are complemented by new permeability and ultrastructural data. The results show the early expression of intercellular junction genes and physiological functionality of their protein products. This, taken together with expression of enzyme and ion transporter genes that are important for CSF secretion, as well as of many other genes whose functions remain to be determined, indicate their role in defining the internal environment of the developing brain. In addition the use of high throughput RNA sequencing of choroid plexus tissue generated substantial new information about expression of influx and efflux transporter genes, the results of which will be published in two companion papers [15], [16].

## Materials and Methods

### Ethics statement

Animal experiments in Melbourne were conducted in accordance with Australian code of practice for the care and use of animals for scientific purposes 7th Edition, published by the National Health and Medical Research Council. All animal research protocols were reviewed and approved by the University of Melbourne Faculty of Medicine, Dentistry and Health Sciences Animal Ethics Committee and registered under ID. Number 1011703. For experiments conducted in Gothenburg, all experiments were approved by the local Ethical Committee at University of Gothenburg (Ethics No. 318-2011) and performed according to the Guidelines for the Care and Use of Laboratory Animals.

### Animal husbandry

Timed-pregnant (embryonic day 15, E15) and non-pregnant adult (6 week, 200–300g weight range) Sprague-Dawley rats were used in this study. These ages were chosen as they have been previously shown to be appropriate for studies of the developing lateral ventricular choroid plexus in rodents [13],[17]. Animals were supplied by the Biological Research Facility at the University of Melbourne (Victoria, Australia) and for the experiments involving electron microscopy rats were bred at the Experimental Biomedicine Animal Facility (University of Gothenburg, Gothenburg; Sweden). For next generation RNA sequencing, lateral ventricular choroid plexuses from E15 (*n = *30) and adult (*n = *30) rats were used. For permeability studies E15 (*n = *16) and adult (n = 15) animals were used to study movement of large molecular (3000Da) biotinylated dextran probes at the light microscopic level, and *n = *4 embryos and n = 2 adults to study to movement of small molecular (286Da) biotin ethylenediamine (BED) at the electron microscopical level.

### Collection of lateral ventricular choroid plexus

The procedure for collection of choroid plexus tissues has previously been described [13]. Briefly, animals were killed by an overdose of inhaled isoflurane (Veterinary Companies of Australia) and brains dissected out under ice-cold RNase-free phosphate buffered saline (PBS, pH 7.3). Both left and right lateral ventricular choroid plexuses were carefully dissected out and placed in fresh ice-cold RNase-free PBS. Plexuses were pooled (*n = *10 animals) and centrifuged at 1000rpm for 30 seconds, excess PBS removed, snap frozen in liquid nitrogen and stored at −80°C. The choroid plexus consists of epithelium as well as blood vessels and mesenchymal stroma. However, the epithelium is the predominant cell type, suggested to represent up to 90% of the plexus tissue [13], [18]. In this study lateral ventricular choroid plexus was taken *in toto*.

### RNA extraction

Total RNA was extracted from pools of E15 and adult lateral ventricular choroid plexus (n = 3 for both ages) using the RNeasy Mini Kit, Qiashredder columns and gDNA removal columns (Qiagen, Valencia, CA, USA) according to standard supplier protocol. Total RNA samples were quantified using a NanoDrop ND-1000 UV-VIS spectrophotometer (Thermo Scientific, Wilmington, DE, USA) and quality checked on an RNA chip using and Agilent 2100 Bioanalyzer (Agilent, Santa Clara, CA, USA). Only samples with an RNA Integrity Number close to 10.0 were kept for further sequencing experiments.

### Illumina next generation RNA sequencing

RNA sequencing was performed at the Australian Genome Research Facility (Melbourne, VIC, Australia). A cDNA library was prepared from 10 µg of total RNA using the mRNA-Seq Sample Preparation Kit (Illumina, San Diego, CA, USA) according to standard manufacturer protocol. Quality of the library was verified using a DNA 1000 chip using the Agilent 2100 Bioanalyzer (Agilent) and quantified by fluorimetry. The library was subjected to 100 bp single end read cycles of sequencing on an Illumina HiSeq 2000 sequencer as per manufacturer protocol. Cluster generation was performed on a c-Bot (Illumina) with a single read cluster generation kit.

#### Data analysis

Short reads were trimmed to remove ambiguous bases from the start and segments with low quality scores from the end, as indicated by the ascii character “B” in Illumina 1.5 phred score encoding. Trimmed reads were mapped with Bowtie version 0.12.7 [19] to the Ensembl rat genome, release 61 [20]. Reads that did not map uniquely were discarded. The number of reads mapped to nuclear genes was determined with HTSeq [21] version 0.4.7p4, using the default “union” counting option. Differential expression between the adult and embryonic samples was detected using an exact test in the Bioconductor [22] edgeR package, version 2.4.0 [23], with common dispersion used to estimate variance between samples. Genes considered significantly differentially expressed were those with a *p*-value of less than 0.05 after Benjamini-Hochberg false discovery rate correction. A combination of gene ontology annotation and manual curation was used to select genes encoding proteins that form part of a cell junction. Gene ontology descriptions for rat were downloaded from Biomart [20], and genes with “junction” mentioned in their gene ontology description were selected. Junction genes of interest were then extracted from this list. Similar searches were carried for other functional categories as described in the Results/Discussion below. For initial analysis genes with >100 sequence reads and age-related fold changes (FC) >2.0 (log_2_FC >1.0) were collated and are summarised in **Tables 1** **,** **2** **,** **3** **,** **4**. For more specific analysis of some particular function categories a lower cut-off of 10 sequence reads was used. Illumina RNA sequencing data have been deposited with the Gene Expression Omnibus (http://www.ncbi.nlm.nih.gov/geo/) under accession code GSE44072.

**Table 1:** Genes associated with choroid plexus differentiation and development.

Gene ID	Log_2_(FC)	*p* value	FC	Gene ID	Log_2_(FC)		*p* value		FC	
(**A**) **Transcripts enriched in E15 embryos**										
*Emx1*	8.2	9.78×10^−46^	302	*E2f3*		3.5		1.17×10^−18^		11
*E2f7*	7.5	7.45×10^−123^	186	*Shh*		2.7		8.27×10^−3^		6.6
*Lef1*	7.0	1.12×10^−71^	130	*Tgfb1i1*		2.4		1.01×10^−18^		5.2
*Nog*	6.6	2.61×10^−53^	95	*Wnt4*		2.3		5.52×10^−17^		5.1
*E2f8*	5.7	1.74×10^−64^	53	*Tgfb1*		2.3		1.21×10^−18^		4.9
*Bmp8a*	5.5	3.77×10^−11^	44	*Wnt5a*		2.1		3.58×10^−18^		4.2
*Irx5*	5.3	1.14×10^−9^	39	*Trim28*		1.9		1.17×10^−16^		3.8
*Gli3*	4.4	1.68×10^−63^	22	*Fzd7*		1.8		6.64×10^−14^		3.4
*Hsf4*	4.2	2.57×10^−14^	19	*Maf*		1.6		1.14×10^−11^		3.0
*Bmp5*	4.0	7.77×10^−46^	16	*Six3*		1.5		1.83×10^−9^		2.7
*Snai1*	3.9	1.13×10^−40^	15	*Ctnnb1*		1.3		7.34×10^−9^		2.5
*Notch1*	3.7	1.53×10^−47^	13	*Smo*		1.0		5.32×10^−5^		2.0
(**B**) **Transcripts enriched in the adult**										
*Bmp15*	3.5	7.47×10^−24^	12	*Sox10*		1.8		7.14×10^−4^		3.4
*Msx1*	2.9	1.04×10^−33^	7.3	*Ppp3r1*		1.5		1.03×10^−10^		2.8
*Igf1*	2.3	5.82×10^−21^	4.9	*Tgfbr2*		1.4		3.76×10^−9^		2.6
*Bmp6*	1.9	9.32× 10^−16^	3.7	*RGD1560225*		1.2		1.64×10^−6^		2.4
*Bmp2*	1.9	3.19×10^−11^	3.6	*Twsg1*		1.1		9.25×10^−7^		2.1

Only transcripts with fold change (FC) >2.0 (log_2_FC >1.0) and *p* value <0.05 are shown above. A comprehensive list is presented in **Table S2**.

**Table 2:** Transcripts of junction-associated genes that were enriched in rat lateral ventricular choroid plexus in embryos (A) or in the adult (B).

Gene ID	Microarray E19	RNA-Seq E15	*p* value	Gene ID	Microarray E19		RNA-Seq E15		*p* value		
**(A) Transcripts enriched in embryos**											
*Col17a1*	8.0	163	2.55×10^−74^	*Cadm3*		-		7.2		4.52×10^−25^	
*Nox4*	-	74	5.63×10^−17^	*Fat1*		-		6.6		1.21×10^−30^	
*Cacng4*	-	43	6.67×10^−48^	*Pvrl2*		1.8		5.0		8.81×10^−23^	
*Palld*	-	38	2.60×10^−73^	*Tmem204*		-		4.5		1.80×10^−17^	
*Pcdh12*	-	34	3.42×10^−57^	*Lpp*		-		4.3		3.91×10^−15^	
*Mip*	-	30	6.93×10^−63^	*Pvrl1*		-		4.2		1.25×10^−16^	
*Cldn8*	-	30	3.63×10^−21^	*Ptpru*		2.5		3.2		1.34×10^−12^	
*Cdh5*	3.5	29	1.79×10^−72^	*Pdlim7*		1.6		3.2		3.00×10^−12^	
*Wnt11*	-	27	1.33×10^−30^	*Arhgef2*		1.3		3.0		1.92×10^−11^	
*Panx1*	1.5	24	9.06×10^−48^	*D3ZXQ2_RAT*		-		2.9		2.29×10^−10^	
*Asam*	12	18	5.25×10^−54^	*PVR*		1.1		2.9		3.93×10^−9^	
*Itga5*	-	18	5.08×10^−54^	*Csda*		2.3		2.9		7.02×10^−11^	
*Amot*	1.4	16	6.50×10^−45^	*Ldb1*		1.3		2.9		6.96×10^−11^	
*Gja4*	5.0	15	1.68×10^−39^	*Amotl1*		1.4		2.8		1.04×10^−9^	
*Ctnd2*	-	15	1.41×10^−42^	*Jup*		2.4		2.7		1.60×10^−9^	
*Cldn6*	8.5	14	2.26×10^−45^	*Jam2*		-		2.6		4.64×10^−10^	
*Col13a1*	-	14	3.21×10^−37^	*Dpp4*		-		2.4		4.49×10^−3^	
*Notch1*	5.3	13	1.53×10^−47^	*Csk*		1.4		2.4		1.67×10^−9^	
*Vangl2*	-	11	5.77×10^−43^	*Ada*		-		2.3		4.01×10^−5^	
*Esam*	-	10	3.25×10^−38^	*Cdh2*		1.2		2.2		1.14×10^−6^	
*Pgm5*	-	8.8	7.57×10^−28^	*Amotl2*		-		2.2		1.43×10^−6^	
*Calb2*	-	8.6	5.90×10^−11^	*Hdac7*		0.9		2.1		6.73×10^−6^	
*Adcyap1r1*	-	8.4	3.24×10^−23^	*Prkd1*		-		2.1		1.46×10^−5^	
*Numbl*	4.0	7.4	1.53×10^−29^	*Ttbk1*		-		2.1		4.51×10^−4^	
*Gja1*	9.7	7.4	2.03×10^−33^	*Baiap2*		1.6		2.1		2.68×10^−5^	
*Itga6*	6.7	7.2	5.45×10^−31^	*Cxadr*		1.7		2.1		9.90×10^−6^	
*Gja5*	3.8	7.2	2.32×10^−20^	*Itgb1*		2.4		2.0		10^−5^	
**(B) Transcripts enriched in the adult**											
*Hnf4a*	-	158	1.94×10^−38^	*Ocln*		0.7		3.4		1.65×10^−14^	
*Aqp4*	3.8	20	5.60×10^−62^	*Dsg2*		-		3.1		4.65×10^−12^	
*Dsp*	2.8	20	6.82×10^−64^	*Vamp1*		1.2		3.1		2.22×10^−9^	
*Cldn2*	2.3	19	3.36×10^−64^	*Cgn*		-		3.1		5.95×10^−12^	
*Cldn19*	1.2	11	4.77×10^−44^	*D4A4T5_RAT*		-		2.9		3.91×10^−10^	
*Cgnl1*	-	9.7	5.68×10^−42^	*Baiap2l1*		1.8		2.7		5.25×10^−10^	
*Trpv4*	2.4	9.1	1.85×10^−39^	*Fh1*		1.3		2.7		1.28×10^−9^	
*Pdzd2*	-	8.4	8.59×10^−37^	*Epcam*		-		2.6		1.20×10^−8^	
*Cldn22*	1.9	6.8	2.80×10^−22^	*Psmb1*		1.5		2.6		4.55×10^−9^	
*Mpp7*	-	6.4	3.18×10^−28^	*Heg1*		-		2.5		1.39×10^−8^	
*Synm*	-	6.0	3.17×10^−27^	*Rhg17*		-		2.4		1.17×10^−7^	
*Pkp2*	1.9	5.8	7.08×10^−25^	*Numb*		2.0		2.3		3.39×10^−7^	
*F11r*	2.1	5.4	1.42×10^−24^	*Cldn9*		2.9		2.3		8.24×10^−6^	
*Tbc1d2*	1.5	4.9	9.07×10^−22^	*Cnst*		-		2.2		1.05×10^−6^	
*Tmem47*	-	4.9	1.47×10^−22^	*Plekha7*		-		2.1		4.08×10^−6^	
*Jam3*	1.0	4.7	1.11×10^−21^	*Adam15*		2.0		2.1		6.92×10^−6^	
*Prkcz*	3.8	4.2	2.00×10^−18^	*Arhgap24*		-		2.0		1.98×10^−5^	
*Dsc2*	0.9	3.7	4.25×10^−15^								

RNA-Seq data are a comparison of E15 and adult. Microarray data are from a previously published study [14] and are a comparison of E19 and adult (hyphens represent transcripts that were not present on the microarray chip). Only transcripts with fold change (FC) >2.0 (log_2_FC >1.0) and *p* value <0.05 are shown above. A comprehensive list is presented in **Table S2**.

**Table 3:** Comparison of cellular adhesion transcripts expressed at the blood-brain and blood-CSF barriers.

Present at plexus	Not present at plexus
*Amotl2*	*Amot*
*Cdh5*	*Amotl1*
*Cgnl1*	*Arhgap17*
*Cldn12*	*Ash1l*
*Dlg1*	*Cldn5*
*Dlgap1*	*Jam4*
*Esam*	*Mpp1*
*F11r*	*Tjap*
*Jam2*	*Tjp2*
*Lin7c*	*Wnk1*
*Magi3*	
*Marveld2*	
*Mpp5*	
*Mpp7*	
*Ocln*	
*Pard3*	
*Pard6g*	
*Scrib*	
*Tjp1*	

Comparison of data presented in the current study on blood-CSF barrier transcriptome and the previously published blood-brain barrier transcriptome [33]. Most (19 of 29) junction transcripts enriched at the blood-brain barrier were present at the blood-CSF barrier. Only one transcript thought to be brain-barrier specific (*Jam4*) was not present at the blood-CSF barrier. Note that our data are from E15 rat choroid plexus, whereas blood-brain barrier [33] are from P2-8 mouse cerebral endothelial cells.

**Table 4:** Identity of ion channel genes.

Channel ID	Type	Gene ID	E15 Enriched Genes (from RNA-Seq)	Adult Enriched Genes (from RNA-Seq)
**K^+^ Channels**				
Kv1.1	voltage-gated	*Kcna1*	*Kcnab3, Kcna5*	*Kcna1, Kcnab1*
Kv1.3	voltage-gated	*Kcna3*	*Kcna3*	
Kv1.6	voltage-gated	*Kcna6*	*Kcna6*	
Kir 7.1	inwardly-rectifying	*Kcnj13*		*Kcnj11, Kcnj13*
Kir3.4	inwardly-rectifying	*Kcnj5*	*Kcnj2, Kcnj3, Kcnj5, Kcnj8*	
Kir1.1	inwardly-rectifying	*Kcnj1*	*Kcnj2, Kcnj9, Kcnj12*	
Kir1.3 (Kir4.2)	inwardly-rectifying	*Kcnj15*	*Kcnj12, Kcnj15*	*Kcnj13, Kcnj11*
TASK1	acid sensitive	*Kcnk3*	*Kcnk2, Kcnk6, Kcnk10*	*Kcnk1, Kcnk4, Kcnk9, Kcn`12, Kcnk15*
Other K^+^	voltage gated		*Kcnb2, Kcnd1,Kcne3, Kcnh3, Kcns3, Kcnq2, Kcnq3, Kcnq4, Kcnv2*	*Kcnd3*
	small conductance Ca^2+^ activated		*Kcnn2*	
**Na+ channels**				
Evidence for involvement of these channels in CP secretion is equivocal. Almost all Na transfer is via transporters				
ENaC	not voltage-gated		*Scnn1g* (very low counts at E15, not detected in adult)	
	voltage-gated		*Scn2b, Scn3a, Scn4a, Scn4b, Scn5a, Scn8a, Scn9a*	
**Ca2+ channels**				
	voltage-dependent		*Cacng4, Cacna2d3, Cacna1i, Cacng8, Cacnb2, Cacna1g, Cacna1h, Cacna1e, Cacng7, Cacna1c, Cacna1a, Cacna1b*	*Cacnb4*
**Receptor-operated channels**				
Purinoceptors	ligand gated		*P2rx1*	*P2ry1,*
**TRP channels**				
	transient receptor potential cation	*Trpv4*	*Trpv2, Trpv6*	*Trpv4*
		*Trpm3*	*Trpc3*	*Trpc1,Trpc2, Trpm3, Trpm7*
**Anion channels**				
VRAC (CLC-2)	voltage-sensitive/volume activated	*Clcn2*		*Clcn2, Clcn4*
**Anion channels not previously identified in choroid plexus**				
CLIC	intracellular C^l−^ channels		*Clic, Clic4*	*Clic2, Clic5, Clic6*
Bestrophins	Ca^2+^ activated Cl^−^	*Best3*		*Best3*
AQP1	water channel, cGMP- activated, non- selective cation channel	*Aqp1*		*Aqp1*
AQP4	water channel	*Aqp4*	*Aqp3*, *Aqp8* (only detectable at E15)	*Aqp4*

Ion channel expression in choroid plexus epithelial cells. Kcnj1 was not detected in RNA-Seq. A comprehensive list is presented in **Table S2**.

### Microarray

Microarray data obtained from E19 and adult Sprague-Dawley rats were obtained from a dataset described in another study [24] with accession number GSE44056. Tissue was collected and processed for microarray in the same manner as that for RNA-Seq (see above). These data are expressed as fold change (compared to adult) in line with the presented RNA-Seq data.

### Physiological experiments

To investigate whether or not movement of passive permeability markers occurs through tight junctions between intimately apposed choroid plexus epithelial cells, E15 and adult rats were injected with biotinylated markers on either the blood side (intraperitoneal injection) or CSF side (intrathecal injection) of the lateral ventricular choroid plexus. For intraperitoneal injections, solutions of biontinylated dextran amine (BDA, MW 3000Da, Molecular Probes/Life Technologies, Grand Island, NY, USA) at a dose of 0.7 mg/g body weight dissolved in 0.9% w/v sterile isotonic saline were made in anaesthetised (inhaled isofluorane) pregnant or non-pregnant rats, using either a glass microcapillary with outer diameter 50–70 µm (E15 embryos) or 30-gauge needle (adults) and left for 40 minutes. In adult animals, a double nephrectomy was performed to halt excretion of the marker in the urine. Intrathecal injections of BDA or BED (Molecular Probes) at a dose of 0.2 mg/g body weight in 0.9% w/v sterile isotonic saline were made using a glass microcapillary (outer diameter 50–70 µm) and left for 10 minutes. A 30-gauge needle was used to first pierce the skull and dura and a small volume of CSF removed (approximately 1.5 µl) before injection. In addition, an incision was made in the dura of the lumbar region of the spinal cord to prevent a pressure rise in the ventricles. For intrathecal injections, the choroid plexus on the contralateral side was investigated to ensure no effects due to surgical damage had influenced the results. Control animals received an injection of 0.9% w/v sterile isotonic saline. The volume of injection did not exceed that of CSF removed.

### Biotinylated dextran amine, light microscopy

Tissue for light microscopy was collected from terminally anesthetised E15 and adult animals (inhaled isoflurane, Veterinary Companies of Australia) and fixed in 4% paraformaldehyde with 2.5% glutaraldehyde in 0.1 M phosphate buffer (pH 7.4) for 3 hours at 4°C. Once fixed, tissue was washed in phosphate buffer (3×10 minutes), embedded in 4% agar and 10 µm sections cut on a vibrating microtome (Leica, Wetlzar, Germany). Sections were floated in phosphate buffer and endogenous peroxidase activity blocked with peroxidase blocking solution (DAKO, Glostrup, Denmark) for 2 hours at room temperature. Sections were then washed in phosphate buffer (3×10 minutes) and stained with an avidin-biotin complex (ABC Kit, DAKO) overnight at 4°C. The following day, sections were washed in phosphate buffer (3×10 minutes) and the ABC reaction was developed with diaminobenzidine (DAB)/nickel staining. One DAB tablet (SigmaFast^TM^ 3,3′-diaminobenzidine tablets, Sigma, St Lois, MO, USA) was dissolved in 1 ml of filtered distilled water with 40 µl nickel ammonium sulphate, with the final solution used to incubate tissue for 10 minutes at room temperature. Following, tissue was moved to a solution containing 1 DAB tablet (Sigma), 40 µl nickel ammonium sulphate and 1 urea hydroxide tablet (SigmaFast^TM^ 3,3′-diaminobenzidine tablets, Sigma) and incubated at room temperature. When a dark precipitate was observed (after approximately 10 minutes) the staining reaction was halted with filtered distilled water. Stained sections were mounted on silanized glass slides with fluorescent mounting media (DAKO) and dried at 4°C overnight.

### Biotin ethylenediamine, electron microscopy

Biotin-ethylenediamine was used as a low molecular tracer (286Da) to assess choroid plexus barrier permeability at an ultrastructural level. The use of this compound as a permeability tracer has been established previously [24]. Pregnant rats at gestational day 15 were anaesthetised and embryos externalized. About 0.5 µL of tracer (0.2 mg/mL in saline) was slowly injected into each lateral brain ventricle of embryos (n = 4) using a fine glass capillary. At 5–8 minutes after the injection, the whole brain was removed and submerged in fixative (2.5% glutaraldehyde in pH7.4 phosphate buffer), before plexuses were dissected out. At the end of the experiment, the mother was killed and tracer solution was directly applied to choroid plexus tissue of the fourth brain ventricle, left for 5 minutes and cold fixative solution slowly applied to plexuses. All plexuses were left in fixative for 2–3 hours in fridge. Tissues were then thoroughly rinsed in phosphate buffer and left overnight in a streptavidin-peroxidase complex (Vector ABC kit PK-6100). The next day, the tissue was stained with a nickel-enhanced DAB reaction (Vector kit SK-4100) according to manufacturer's specifications. Tissue was then processed as for conventional electron microscopy with osmium, dehydrated through increasing concentrations of ethanol and embedded in araldite–epon. Sections were cut using a Reichard Ultracut E microtome and examined under a LEO 912AB electron microscope. Control tissue was collected from un-injected embryos, was processed as above but gave no visible reaction product.

### Immunohistochemistry

Sagittal sections through E15 and adult rat brain including lateral choroid plexus were selected from the collection of rat tissue at the Faculty of Health and Medical Sciences, University of Copenhagen, for immunohistochemical detection of carbonic anhydrase-related protein VIII (CA8), SLC4A1 and CLIC3. Sections were deparaffinised in xylene, rehydrated through graded alcohols followed by treatments in 0.5% hydrogen peroxide in methanol for 15 min and rinsing in Tris-buffered saline (TBS) as described previously [13]. Following removal of non-specific binding, sections were incubated in primary antibodies (SLC4A1, 8566-1-AP, 1:400; CLIC315971-1-AP, 1:200; or CA8, 12391-1-AP, 1:150. All antibodies were obtained from Proteintech, Manchester, UK). After overnight incubation sections were washed in TBS and incubated for 30 min in EnVisionTM+ System/HRP (DAKO), K5007. This was followed by 6 min incubation with DAB-chromogen solution (DAKO) and counterstaining with Mayer's haematoxylin, dehydrated and mounted with DPX. Control sections contained no primary antibodies and were always blank.

### Photography and image preparation

Digitized images were obtained using an Olympus DP70 camera housing (Olympus, Tokyo, Japan) attached to an Olympus BX50 light microscope (Olympus). A 10× eyepiece and 40× objective lens were used. Raw image files were process in Adobe Photoshop CS5® (Adobe ® Systems, San Jose, CA, USA). The brightness and curve functions were used to obtain images with background close to white. There was no other manipulation of images.

## Results and Discussion

Sequencing of total RNA samples collected from E15 and adult lateral ventricular choroid plexus was completed using the Illumina platform. A total of 29479 transcripts were mapped, of which 5872 transcripts were enriched at E15 and 4781 enriched in the adult. Over 18000 (approximately 60%) of these genes were already expressed at adult levels in E15 samples, suggesting a great deal of maturity of expression levels in this tissue. Refer to **Table S1** (raw transcript count) and **Table S2** (summarised analysed data) for full dataset.

### Genes associated with choroid plexus differentiation and development

Several genes previously associated with neural development were detected in the rat lateral ventricular choroid plexus epithelium. This included many transcription factors traditionally associated with the development of neuroependymal cells of the ventricular wall at the root of the choroid plexus that control differentiation into epithelial cells of the plexus proper. Some, such as *Ngn2* and *Foxg1* oppose the formation of choroid plexus epithelial cells, instead causing cells to become neural in nature and we did not detected these transcripts in plexus epithelium. Others such as *E2f5, Pcna* and members of the *Hes* family actively promote the conversion into plexus epithelial cells [25], [26]. Proteins for these transcripts are localised both in the neuroependyma and in the choroid plexus epithelial cells themselves (these transcripts did not display altered expression between E15 and adult, see **Table 1**). Many genes traditionally associated with developmental milestones were detected in the choroid plexus epithelium: *Gli3* and *Emx* have been shown to be important for dorsal telencehpalic development [27]. These transcripts were detected and enriched in the E15 plexus 22-fold (*Gli3*), 302-fold (*Emx1*) and 3.6-fold (*Emx2*).

Mice deficient in TWSG1 have abnormal forebrain growth manifesting as holoprosencephaly during its development, however, the expression and potential roles of *Twsg1* in postnatal brain development are less well understood. We found that *Twsg1* was expressed in the rat choroid plexus epithelium, enriched 2.2-fold in the adult. Previously, *Twsg1* has been reported in the hippocampus and other brain regions with the strongest expression observed in choroid plexus in embryos and adult [28]. In addition *Bmp3* (detected at E15 only), *Bmp5* (16-fold), and *Bmp8a* (44-fold) were enriched in the embryo whereas *Bmp2*, *Bmp6* and *Bmp15* were enriched in the adult – see **Table 1**. BMP antagonists CHORDIN (*Chrd*) and NOGGIN (*Nog*) have previously not been detected in choroid plexus epithelium, however we report them as enriched 2.3- and 95-fold at E15 respectively.

Another set of transcription factors, *E2f5*, *Foxj1* and *P73*, cause non-obstructive hydrocephalus in mouse when expression is altered [29]. These factors are present in choroid plexus epithelial cells, immediately after their differentiation from the neuroependyma. In the case of *E2f5*, levels of the protein in the brain are highest during mouse embryonic development and lower in the adult [29], [30]. This correlates well with the current data, with several members of the transcription factor E2F family of proteins enriched at E15 in the rat plexus epithelium (see **Table 1**). The expression of E2F proteins is strongest in choroid plexus epithelium of both mouse and human earlier in development; however it may be more important for the maturation of choroid plexus epithelial cells rather than for their original development from neuroependymal cells [29].

Lymphoid enhancer binding factor 1 (*Lef1*) was enriched 130-fold at E15 in the current study and has been identified by *in situ* hybridisation as early as E13.5 in lateral ventricular choroid plexus of mouse embryos [31]. Insulin-like growth factor (IGF1) is synthesised by the choroid plexus epithelial cells and is secreted into the CSF. In embryonic mice, IGF1 levels in the CSF decreased from days E12 to E15, increased rapidly from days E16 to E18 and then decreased from days E19 to E21 [32]. Our current study it was enriched 4.9-fold in the adult. Both *Lef1* and *Igf1* transcript were reported as highly enriched in brain endothelial cells compared to liver and lung [33], suggesting their importance in the development of central nervous system cell types. As IGF1 is a potent natural activator of the protein kinase B signalling pathway, the results suggests its increased expression in embryonic plexus cells may be acting to aid in cell growth and proliferation, at a time when plexus growth is extremely rapid [13], [17].

The extent of expression of developmental genes in the choroid plexus is not fully realised in this study, however the paucity of research on plexus development makes these insights of great importance. Most significantly, the equilibrium between transcripts generally thought to reside only in neuroependymal cells, and not plexus epithelium is of great interest (e.g. *Twsg1*, *Shh*, *Bmp* family, refer also to **Table S2**). Their expression ensures proper establishment of the choroid plexus, which is increasingly recognised as an important organiser of brain development as it is intimately linked to the dorsal neural tube through a shared boundary organiser [34], [35]. Thus, without proper development and functioning, not only is the internal milieu of the brain compromised, normal brain development itself is hindered.

### Junction-associated genes

**Figure 1:**
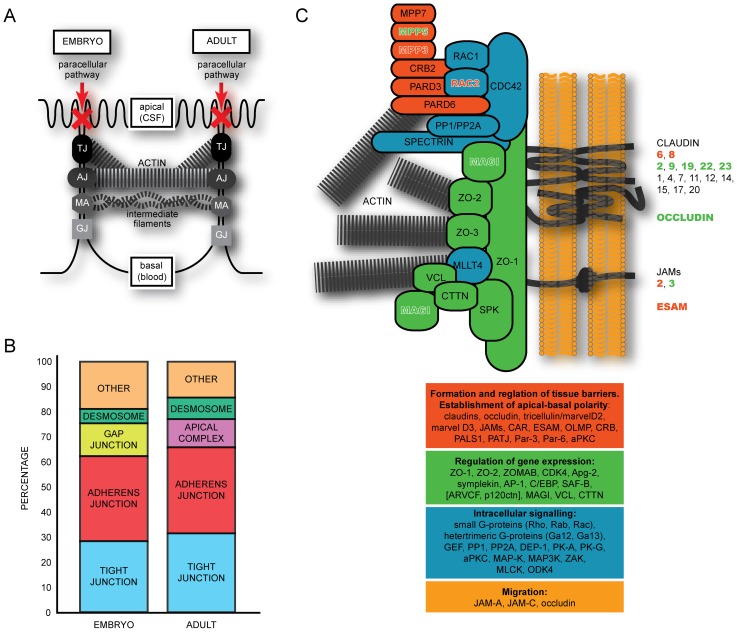
Proteins involved in the formation of junctions between intimately apposed cell membranes. **A:** The main barrier function of the epithelium depends on tight junctions, which are connected with the actin cytoskeleton (as are adherens junctions). Other types of junctions such as macular adherens (desmosomes) and gap junctions also play a role. There general structure and permeability of these junctions does not change throughout development. **B:** Proportion of gene transcripts with enriched expression identified for different components of intercellular junctions (genes with unchanged levels of expression are not represented). Note the lack of enrichment of transcripts involved in apical complexes in the embryo, and gap junctions in the adult. **C:** Three types of transmembrane protein are part of tight junctions: occludin, claudins and junctional adhesion molecules (JAMs) which are connected to adaptor proteins such as ZO1, ZO2 and ZO3. Many additional proteins are also essential, including PAR3, PAR6 and aPKC. This complex, which is important for cell polarity, is regulated by Cdc42, as is the CRUMBS3–PALS1–PATJ-complex, which is essential for tight junction assembly. The precise role of RhoA and Rac in tight junction regulation has still to be elucidated. Recently, it was suggested that RhoA-dependent phosphorylation of occludin is crucial for tight junction function. From RNA sequencing experiments we found that targets for several junctional proteins were up-regulated in the embryo (red text), some were up-regulated in the adult (green text), while a large proportion did not alter expression levels (black text). Expression values are presented in **Tables 2** and **S2**. Abbreviations: Apg-2, ATP and peptide-binding protein in germ cells; aPKC, atypical protein kinase C; ARVCF, armadillo repeat gene deleted in velo-cardio-facial syndrome; CAR, Coxsackie and adenovirus receptor; Cdc42, cell division cycle 42 (GTP binding protein); CDK-4, cyclindependent kinase-4; C/EBP, CCAAT/enhancer binding protein; CLMP, Coxsackie- and adenovirus receptor-like membrane protein; CRB-2,-3, human crumbs homologue-2,-3; DEP-1, density-enhanced phosphatase-1; ESAM, endothelial cell-selective adhesion molecule; GEF, GDP/GTP-exchange factor; JAM, junctional adhesion molecules; PALS1, protein associated with Lin-7; PATJ, Pals1 associated tight junction protein; MAP-K, mitogen-activated protein kinases; MLCK, myosin light chain kinase; Par-3, -6 partitioning defective protein-3, -6; PK-A, -G protein kinase-A, -G; PP1, protein phosphatase1; PP2A, phosphatase2A; Rac, ras-related C3 botulinum toxin substrate; Rho, Rhodopsin; SAF-B, scaffold attachment factor-B; ZAK, ZO-1 associated kinase; ZO-1, -2, -3, Zonula occludens protein-1, -2, -3; ZONAB, ZO-1-associated nucleic acid binding protein.

Choroid plexus epithelia are one of the interfaces that separate the central nervous system (CNS) from the periphery. They are the site of barriers as well as sites of exchange of ions and molecules from and into the CSF (and thence the brain). The intercellular junction complexes between adjacent cells include gap junctions, desmosomes, adherens junctions and tight junctions (**Fig. 1A**). Such junctions are essential for the barrier properties of the choroid plexus and important for normal brain development. Gap junctions, comprise intercellular channels in the plasma membrane of adjacent cells allowing diffusion of small molecules between cells; desmosomes connect the plasma membrane to intermediate filaments in the cytoplasm; adherens junctions connect the actomyosin cytoskeleton with the plasma membrane; and tight junctions form bands of close contact between adjacent cells usually around the cell's apical surface and are highly ordered membrane contact sites or ‘kissing points’, comprising a network of intra-membrane fibrils [36]. Each of these junction classes are multiprotein complexes that mediate cell-cell adhesion, with tight junctions determining important features of epithelial cell permeability. The combination of tight junctions, desmosomes and adherens junctions, collectively provide a barrier that prevents or largely limits molecules from diffusing between cells (paracellular movement). Recent evidence also indicates that tight junction proteins play an important role in the establishment and maintenance of apical-basal [37].

Analysis to extract transcripts involved in all four cell-cell junction types using Gene Ontology categories produced a list of 448 transcripts. Only those transcripts with a raw count number above 10 and *p*<0.05 were considered for further analysis (a total of 186 genes). From the list of transcripts, manual removal of false positives (with non-junction protein products) by examination of biological function showed 54 transcripts were enriched at E15, while 35 were enriched in the adult (**Table 2**). It is important to note that many more transcripts were found expressed at levels equal in both the embryo and in the adult – suggesting their already mature level of expression at embryonic day 15 (see **Table S3**). At E15 those transcripts with protein products with known involvement in intercellular junction complexes that were enriched compared to the adult were represented by tight junctions (15 transcripts), adherens junctions (18), gap junctions (7), desmosomes (3) and other junctional genes (10). In the adult no transcripts were enriched that were associated with gap junctions, though there were several involved in tight junctions (11), adherens junctions (12) desomosomes (3), apical junction complexes (4) and other junctional genes (5), see **Fig. 1B**. These data suggest that gap junction coupling and intracellular communication is of great importance in the embryonic choroid plexus epithelium. Overall the study adds substantially to previously published transcriptome information on plexus epithelial cell junctions. We previously identified 14 of these genes that were expressed at a higher level at E15 and 8 in adult mouse choroid plexus, in a microarray study of mice [13]. Of the claudin genes identified in the present study (**Table 2**) *Cldn3* and *Cldn6* are reported to be expressed at higher levels in embryonic (E19) rat lateral ventricular choroid plexus (2.6- and 8.5-fold respectively [14]) whereas *Cldn2*, *Cldn9* and *Cldn22* were expressed at higher levels in the adult (2.3-, 2.9-1.9-fold respectively) as in the present study (**Table 2**).

Some genes in the cellular junction category were already expressed in the embryo and did not change their expression level by adulthood (e.g. gap junction proteins *Gjb4, Gjb5, Gjd2*). There were however, age-related variations in expression between the two ages, which will be the focus of this description. **Fig. 1** and **Tables 3** and **S3** summarize all these data (**Table 2** includes data from the Affymetrix genechip part of the study). Cellular junction gene expression has been relatively little studied in the choroid plexus, particularly in development. Some studies have looked only at a select few claudins in the adult mouse using a combination of freeze fracture electron microscopy and immunofluorescence [38], [39], or more broadly using genechip analysis of adult [40] or embryonic and adult mice [13]. A recent study has described more extensively the claudins and other tight junction-associated proteins in rat choroid plexuses from late embryonic to adult stage [14]. These previous studies provide valuable insights into the junction-associated proteins likely present at the blood-CSF barrier, however no study to date has provided a comprehensive list in both the embryo and adult. **Table 2** and **Fig. 1** summarize the main junction-related genes identified in the present study, for both RNA-Seq and Affymetrix results. **Fig. 1** indicates where the various genes are thought to be located in the different components of intercellular junctions, based on previous published data that were mainly from *in vitro* experiments (for reviews see [41]–[43]. Many of the genes identified in the present study have not previously been described in the choroid plexus.

Occludin (*Ocln*) has previously been identified in tight junctions of developing rat, with similar levels of expression in embryonic (E19) and adult choroid plexus [14]. Here we also report its expression in the epithelial cells of the choroid plexus, with an adult expression level of 3.4-fold higher than at E15. In total, transcripts for 16 claudins (*Cldn*) were present in the embryonic and adult choroid plexus, with 9 unchanged, 5 enriched in the adult and 2 at E15. Transcripts for *Cldn8* (30-fold) and *Cldn6* (14-fold) were expressed at much higher levels at E15 than in the adult, whereas *Cldn2* (19-fold), *Cldn19* (11-fold) *Cldn22* (6.8-fold) and *Cldn9* (2.3-fold) were expressed at higher levels in the adult plexus. At E19, *Cldn2* (19-fold), *Cldn19* (11-fold) and *Cldn22* (6.8-fold) level of expression were already close to adult level. *Cldn23* was enriched 4.3-fold in the adult plexus, however its actual expression level was much lower than the other reported enriched transcripts. We did not see definitive expression of *Cldn3* or *Cldn5*by RNA-Seq analysis. CLAUDIN-5 has been reported to be present in the endothelial cells of blood vessels penetrating into the choroid plexus stroma but absent from the epithelial cells [14]. Its absence in our study is an important indication that the RNA-Seq screen was not picking up contaminants from non-epithelial cells in the plexus (see also Limitations of study, below). CLAUDIN-6 is considered a marker for early epithelialization [44] and is clearly involved in barrier formation, which was particularly shown for the epidermal barrier [45]. This also validates our methodology, which shows at both E15 and E19 the early expression of *Cldn6* in the plexus. Claudin family members *1*, *4*, *7*, *11*, *12*, *14*, *15*, *17* and *20* were present with no change in their expression level between E15 and adult. CLAUDIN1 protein has previously been identified in plexus tight junctions [46]. Our finding of *Cldn1* expression at similar levels in embryonic and adult plexus confirms the recent report in both rat and human developing choroid plexus, using a combination of qRT-PCR, western blotting and immunohistochemistry [14].

The substantially higher expression of *Cldn6* and *Cldn8* in the embryo suggests that they are key claudins expressed at the blood-CSF barrier in early development and likely to be involved in the functional impermeability of plexus tight junctions early in brain development [47]. In comparison, *Claudin-2*, -*9*, -*19*, -*22* and -*23* are up-regulated in the adult and either reinforce the barrier properties of the tight junctions or perhaps reflect other currently undefined functions. *Cldn3* expression in the choroid plexus epithelium was demonstrated by Kratzer and colleagues [14] but this gene was not identifiable in the RNA-Seq data set (see Limitations of Study, below); however *Wnt3a* expression was present (only at E15) and has been shown to be involved in formation of the blood-brain barrier by up-regulating *Cldn3* expression [48]. It may be that *Wnt3a* or different Wnts (e.g. *Wnt11*, 28-fold enriched at E15) are having a similar effect on different CLAUDINs, causing the initial formation of tight junctions at this interface.

In general, the differentially expressed *Cldn* family members belong to the barrier type as well as to the channel/pore-forming type. CLAUDIN-8 interacts with CLAUDIN-4 to form a paracellular Cl^−^ channel [49]. On the other hand it acts as a cation barrier increasing the transepithelial electrical resistance of kidney epithelial cells and may replace CLAUDIN-2 when overexpressed in kidney cells [50]. CLAUDIN-6 down-regulation may contribute to the malignant progression of certain types of breast cancers [51]. However, since it is known that the permeability properties of a tissue is determined by a distinct combination of several claudins, no further conclusions can be drawn based on down-regulation of single claudins.

Of particular interest is the differential expression of the hepatocyte nuclear factor 4α (*Hnf4α*), a member of the nuclear receptor superfamily necessary for metabolic functions in liver and for proper insulin secretion in the pancreas [52]. Here we have shown that *Hnf4α* is highly enriched in the adult choroid plexus. In earlier studies *Hnf4α* was shown to regulate the expression of numerous genes encoding junction- and adhesion-related proteins during embryonic development of the mouse liver. These include proteins involved in the formation of adherens junctions, tight junctions, desmosomes, and gap junctions, as well as proteins involved in epithelial polarization, cytoskeletal organization, and signal transduction [53], [54]. It was further shown that *Hnf4α* directly binds to regulatory elements within many of these genes [54]. Some of the junctional genes, known to be induced by *Hnf4α*, indeed show higher expression in the adult choroid plexus. These include claudin-2 (*Cldn2*), F11 receptor (*F11r/JAM1*), occludin (*Ocln*), desmocollin (*Dsc2*), and plakophilin2 (*Pkp2*). In addition, HNF4α plays a role in controlling the expression of drug transporters by binding to regulatory sequences of ABC transporters (ABCB4 and ABCC1) as shown in Caco-2 cells [55]. Although experimental evidence is still lacking it may be assumed that the marked up-regulation of *Hnf4α* expression in the adult choroid plexus is due to the establishment of the mature blood-CSF barrier.

We also report that several gap junction genes were present in the choroid plexus and expressed at a higher level at E15; these included *Panx1*, *Gja1, Gja4*, *Calb2*, and *Csda*. Their expression decreased to some extent at E19 and of these only *Gja1* and *Csda* were expressed at substantial levels in the adult. There were also several genes were expressed at one age only – such as the gap junction protein genes *Gjd2*, *Gjb5*, *Gje1* and *Gjb4* in the embryo (see **Table S3**). Extensive gap junctions have been reported to be present in developing choroid plexus, but are largely absent in the adult [56] reflecting the importance of intercellular communication between the epithelial cells of the developing plexus.

Adherens junctions are an important component of junctional complexes between epithelial cells. In this study we identified 18 adherens or adhesion genes that were expressed at a higher level in E15 choroid plexus (**Table 2**). In comparing E15 and adult choroid plexus junctional genes, more tight junction and adherens/adhesion junction genes were expressed at a higher level in the embryonic plexus than in the adult; genes for the apical junction (4) were expressed only in the adult (**Table 2**).

Expression levels for most cytoplasmic/regulatory adaptors were similar at the two ages (e.g. *Mllt4*, *Rac1*, *Cdc42*, *PP1/PP2A*). Of the small number of genes with altered expression levels, the junctional adhesion molecules, *Jam1* (F11r) and *Jam3*, were enriched in the adult (5.4- and 4.7-fold respectively); this is a novel plexus-specific discovery. In contrast, *Jam2* was enriched at E15 (2.7-fold). Blood-brain barrier endothelial cell-specific *Jam4* [33] was not seen in plexus epithelial cells. *Marveld2* was also expressed at both ages in low levels. MARVELD2 protein has been shown to seal tight junctions at sites where more than two cells meet [57], [58]; since its distribution is limited to such sites, even low expression levels suggest that it may be significant.

Additionally, *Amotl2* (angiomotin-like protein 2) transcript was seen slightly enriched (2.2-fold) in the embryo. AMOT plays a central role in tight junction maintenance via the complex formed with ARHGAP17, which acts by regulating the uptake of polarity proteins at tight junctions. Together they appear to regulate endothelial cell migration and tube formation and may also play a role in the assembly of endothelial cell-cell junctions [59]. Another component of the epithelial apical junction complex, *Cxadr* (∼2-fold enriched at E15 and E 19), is essential for tight junction integrity and has been shown to recruit other junction proteins (e.g. MPDZ) to intercellular contact sites. In addition to *Amotl*, *Cdh5* and *Esam* were expressed at higher levels in E15 choroid plexus (29- and 10-fold respectively). All three of these genes have previously been associated with tight junction formation in cerebral endothelial cells; their presence here may indicate contamination by blood vessels in choroid plexus stromal tissue or alternatively a new finding relevant to choroid plexus development (see also Limitations of study, below).

Finally, the transmembrane collagen XVII (*Col17a1)* was shown to be highly expressed at E15 and remained high at E19, being 163-fold and 8-fold higher than in the adult choroid plexus, respectively. There is a recent report showing that the collagen XVII is a component of the podocyte filtration barrier in the kidney glomerular basement membrane [60]. A possible involvement of this transmembrane collagen in the embryonic blood-CSF barrier remains to be studied).

Here we have provided an exhaustive list of junction transcripts at the embryonic and adult choroid plexus of the rat. We report on a small number of transcripts that alter expression throughout development, however find that most are already expressed at adult levels by E15, as outlined above. Comparing expression of junction related genes at the blood-CSF barrier to that in the blood-brain barrier interface [33], approximately 65% (19 of 29) junction proteins highly expressed at the blood-brain barrier were expressed at the blood-CSF barrier (see **Table 3**). Of the transcripts highlighted by to be highly enriched in blood-brain barrier endothelial cells over peripheral endothelial cells from the liver and lung (*Cgnl1*, *Marveld2*, *Mpp7*, *Ocln*, *Pard3* and *Jam4*, see [33]), only transcript for *Jam4* was not detected in choroid plexus epithelial cells (at any age).

We have not attempted to draw correlations between these individual transcripts, nor discuss their interactions. For many junction proteins we do not know much about their function, nor how important the interactions between them and cytoskeletal anchoring proteins are. Moreover, due to the large number of associations between individual components, it is unlikely that all interactions will occur at the one time. Recent results suggest that the proteins that form junctions in fact form subcomplexes [61], [62]. Large-scale RNA sequencing studies provide vast amounts of information pertaining to the presence of individual transcripts; however they provide no information about interactions – particularly important for junction assembly and function. Functional analysis of particular junctional components will likely rely on RNA interference studies, likely requiring the manipulation of several components simultaneously [36].

### Mechanism of CSF secretion: Enzymes, ion channels and ion transporters

Of key importance to the developing and adult brain is the stability of the ionic environments of the CSF. The ionic composition of this fluid that bathes the brain and fills the ventricular system is different between the embryo and the adult [63], [64]. It is known that the CSF/plasma gradients for some ions are established very early in development – something that would be impossible without the presence of functional tight junctions (see [5] for review). The presence of ion channel and transporter transcripts at high levels in the E15 rat lateral ventricular choroid plexus (see **Table S4**) confirms that the integrity of plexus tight junctions is effective and are already restricting movement of ions through the paracellular pathway; this also suggests that ion pumps in the plexus epithelial cells are already active.

It has been known for many years that the principal drivers of CSF secretion are intracellular carbonic anhydrase and Na/K-ATPase in the apical membrane of choroid plexus epithelial cells [65] with various ion channels in the basolateral and apical membranes of the plexus epithelial cells also making an important contribution. The molecular basis of CSF secretion in adult choroid plexus epithelial cells has been studied extensively [66]–[70]. The key mechanisms identified to date have been reviewed by Brown et al. [66], Millar et al. [67] and Damkier et al. [71].

#### Ion channels. Fig. 2

**Figure 2:**
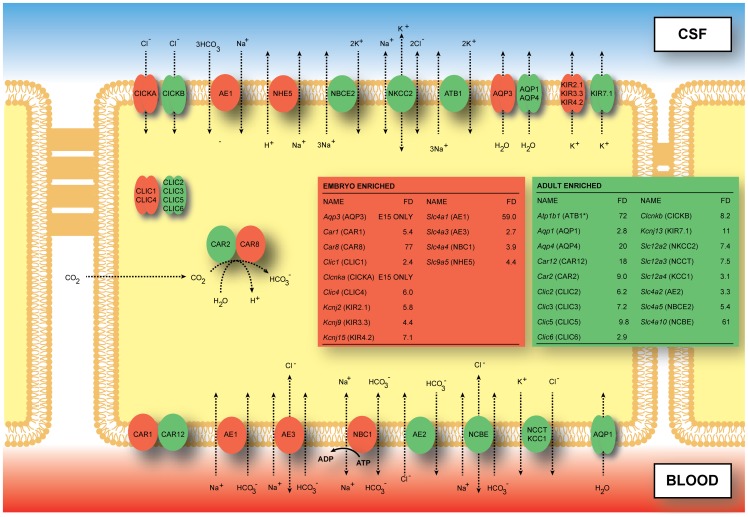
Localization of proteins for ion transporters, channels and associated enzymes and identification of their corresponding genes in adult and immature rat choroid plexus. Data for the localisation of the proteins are from a previous study [100] and see also **Tables 3** and **4**. CSF secretion results from coordinated transport of ions and water from basolateral membrane to cytoplasm, then sequentially across apical membrane into ventricles (for review see [65]). On the plasma-facing membrane is parallel Cl^−^/HCO_3_^−^ exchange (AE2, *Slc4a2*) and Na^+^/HCO_3_^−^ co-transport (NBC1, *Slc4a4*) with net function bringing Cl^−^ into cells in exchange for HCO_3_^−^ [101]. Also basolaterally located is and Na-dependent Cl^−^/HCO_3_^−^ exchange (NCBE, *Slc4a10*) that modulate pH and perhaps CSF formation [102]. Apical Na^+^ efflux by NHE5 (Slc9a5) and ATB1 (*Atb1b1*, Na^+^/K^+^-ATPase, asterisk) maintains a low cell Na^+^ that sets up a favorable basolateral gradient to drive Na^+^ uptake [103]. Na^+^ is extruded into CSF mainly via the Na^+^/K^+^-ATPase pump (ATB1, *Atb1b1*) and, under some conditions, the Na^+^/K^+/^-Cl^−^ co-transporter NKCC2, *Slc12a2*, see [104] for review). Overall cell volume is maintained by the K^+^/Cl^−^ co-transporters NCCT (*Slc12a3*) and KCC1 (*Slc12a4*). Aquaporin (AQP1/3/4) channels on CSF-facing membrane mediate water flux into ventricles [105]. Polarized distribution of carbonic anhydrase (CAR) and Na^+^/K^+^-ATPase, and aquaporins, enable net ion and water translocation to CSF (see [104], [106] for review). The gene *Slc4a7* (NBCn1) was not detected by RNA-Seq, although it has been reported in both rat and mouse choroid plexus [107]); this may have been for technical reasons or because of lack of antibody specificity see section “Limitations of study”. The genes for *Clir* (chloride inwardly rectifying) channels has not been previously identified but are probably *Clica* and *Clicb*. The gene for VRAC (volume regulated anion channels) is not known [108]; see also **Table 4**. The carbonic anhydrases CAR2 and CAR8 have an intracellular distribution; CAR8 has been shown to lack the characteristic enzyme activity of these proteins [73]. It is not known whether it is functional in the embryo. The CLIC chloride channels are also intracellular [109] and as such we have placed them cytosolically, however it is more likely that they sit on the internal membrane of the cell and aid in movement of Cl^−^ to other channels. The inset boxes show the fold differences for genes expressed at a higher level in the embryonic (red) or in the adult (green) choroid plexus.

summarizes the ion channel and transporter genes and their cellular/membrane localisation in adult choroid plexus epithelial cells. The insets indicate the NCBI gene nomenclature, together with other ion channel and transporter genes identified in the current study that have not previously been described in choroid plexus. **Fig. 2** also illustrates ion channel and transporter genes identified in E15 choroid plexus and the comparative expression levels of these genes at the two ages studied. **Tables 4** and **5** list the channel and transporter proteins and their genes identified in E15 and adult choroid plexus that are known to be functional in adult choroid plexus [67], [71]. In the RNA-Seq analysis of adult rat choroid plexus there were 26 ion channel genes expressed at higher levels than in the embryonic choroid plexus (see **Table S4**). Of these, 15 were K^+^ channels (fourteen voltage-gated channels and one *Tmem* channel transcript) two Ca^2+^ and four Cl^−^ channels four transient receptor potential cation channel (*Trp*) genes and one voltage dependent anion-selective gene (*Vdac1*). More than twice as many genes for channel proteins (59) were expressed at a higher level in the embryonic plexus than in the adult (**Fig. 2** and **Table S4**). Twenty-four *Kcn* gene family members for K^+^ voltage gated channels were expressed levels of between 2- and 210-fold in E15 choroid plexus, together with two *Kct* channel genes. Thirteen genes of the *Cacn* family of subunits of voltage gated Ca^2+^ genes were expressed at higher levels in E15 choroid plexus than in the adult, as were seven *Scn* genes (voltage gated sodium channels), nine Trp channels, two Cl^−^ channel genes (*Clic1*, *Clic4*) and two cyclic nucleotide gated channels (*Cnga1* and *Cnga3*). In addition there were 6 ion channel genes that were expressed only at E15 (*Kcnmb2*, *Tmc5*, *Clcnka*, *Scnn1g*, *Kcnh8*, *Kcnmb1*) and one in the adult (*Kcnk9*) (**Table S4**).

**Table 5:** Identity of ion transporter and associated enzyme genes.

Protein ID	Transport Function	Gene ID	E15 Enriched Genes (from RNA-Seq)	Additional Genes with Unaltered Expression (from RNA-Seq)	Adult Enriched Genes (from RNA-Seq)
Na^+^/K^+^ ATPase	Na^+^/K^+^	*Atp1b1*		*Atp1a3*	*Atp1a1, Atp1b1, Atp1b2*, *Atp1b3*
Carbonic anhydrase 2	H^+^/HCO_3_^−^ synthesis	*Car2*	*Car1, 8, 10*	*Car6, 9*	2, 3, *4*, *5b*, *12*, *13*, *14*
NKCC1	Na^+^/K^+^/Cl^−^	*Slc12a2*			*Slc12a2*
NBCe2	Na^+^/HC0_3_^−^	*Slc4a5*			*Slc4a5*
NHE1	cation proton antiporter	*Slc9a1*	*Slc9a5* (NHE5)	*Slc9a1*	*Slc9a6* (NHE6), *Slc9a7* (NHE7)
NaBC1	sodium borate/HCO_3_^−^/anion	***Slc4a11***	*Slc4a3*, *Slc4a4*		
NBCn1	Na^+^/HCO_3_^−^	*Slc4a7*	*Slc4a4*		
NCBE/NBCn2	Na^+^/HCO_3_^−^/Cl^−^	*Slc4a10*			*Slc4a10*
NCC	Na^+^/Cl^−^				*Slc12a3*
	Na^+^/K^+^/Ca^2+^				*Slc24a3*
KCC3	K^+^/Cl^−^	***Slc12a6***			*Slc12a4* (KCC1)
KCC4	K^+^/Cl^−^	*Slc12a7*		*Slc12a7*	*Slc12a4* (KCC1)
AE2	Cl^−^/HCO_3_^−^	*Slc4a2*			*Slc4a2*
AE1	Cl^−^/HCO_3_^−^	*Slc4a1*	*Slc4a1*		

**Figure 3:**
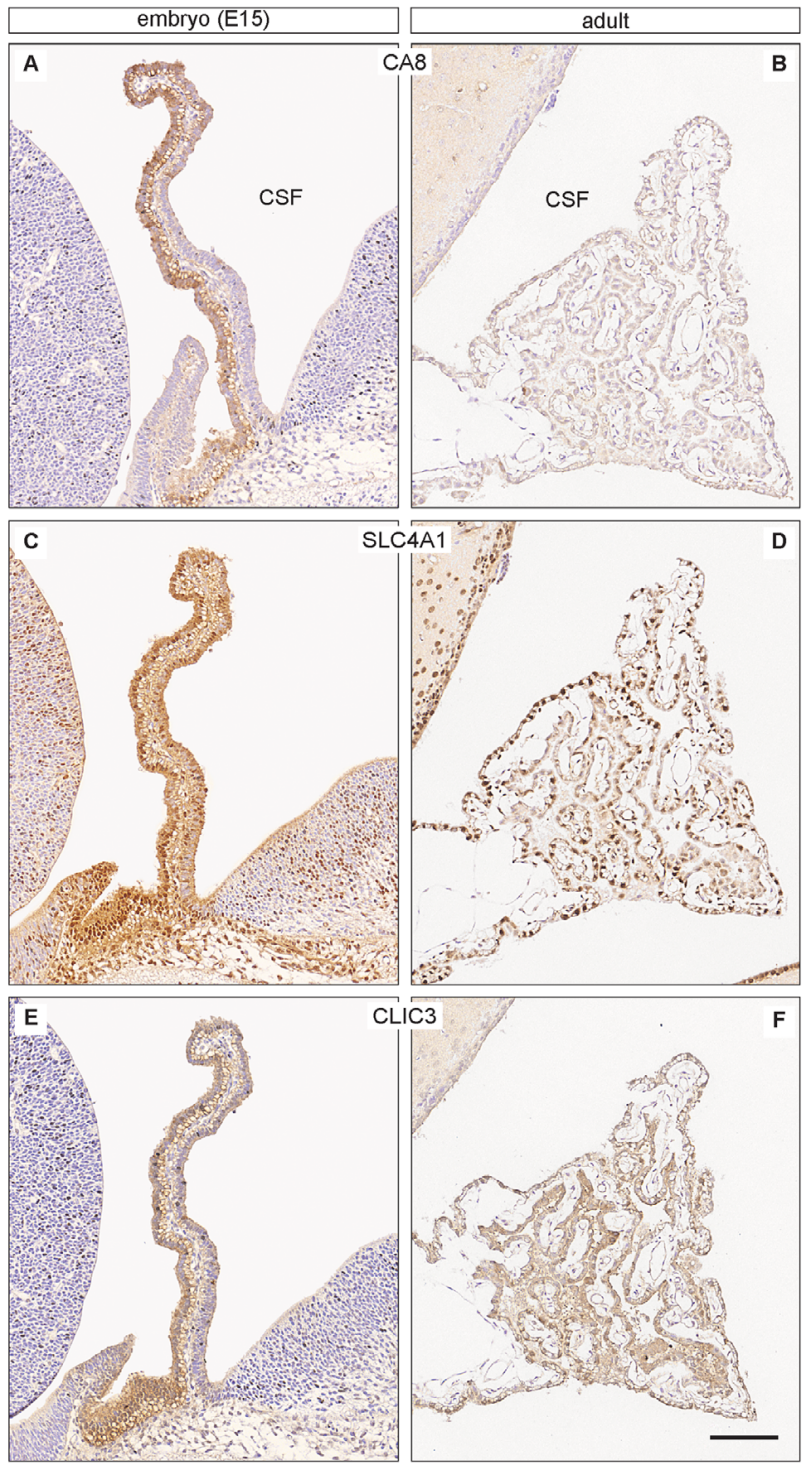
Immunohistochemical localisation of CA8, SLC4A1 and CLIC3 gene products in embryonic (E15) and adult lateral choroid plexus. Embryonic choroid plexus (**A**, **C**, **E**) is compared to that of adult (**B**, **D**, **F**). Note the differences in patterns of immunoreactivity between the ages. Embryonic plexus epithelium is strongly stained for CA8 while virtually no reactivity was detected in the vasculature (**A**). Adult plexus is unstained (**B**). SLC4A1 immunoreactivity is pronounced in E15 plexus where all cell nuclei and cytoplasm in both epithelium and endothelium are strongly positive (**C**), whereas the immunoreactivity in the adult plexus is varying but mainly nuclear in both epi- and endothelium (**D**). Following staining for CLIC3 a marked reaction is observed in the entire embryonic plexus (**E**) in contrast to that in the adult where vasculature is devoid of staining and the cytoplasm of scattered choroid plexus epithelial cells is strongly immunoreactive (**F**). Scale bars: 100 µm for all. Abbreviation: CSF, cerebrospinal fluid.

Enzymes and ion transporters known to be involved in CSF secretion. Protein ID from [72]; these ion transporters have been shown to be active in adult choroid plexus; as an example the cellular localization of SLC4A1 is shown in **Fig. 3C, D**. Gene ID and function from NCBI (http://www.ncbi.nlm.nih.gov/gene). Genes in bold were not detected in either RNA-Seq or Affymetrix datasets. *Slc4a7* was only identified in Affymetrix. Slc12a2 (NKCC1) has previously been identified in embryonic mouse choroid plexus as has *Slc12a4* (KCC1) [110] and *Slc4a10* (NCBE) [111]. Genes underlined have not previously been identified in rat choroid plexus (but cf. with mouse choroid plexus [13]). A comprehensive list is presented in **S2**. Most of the genes listed here were identified in both RNA-Seq and Affymetrix datasets.

The data in **Fig. 2** and **Table 4** show that all but one of the K^+^ channel genes (*Kcnj1*) known from physiological studies in adult choroid plexus were identified in the RNA-Seq screen, but only two of these were expressed at a higher level in the adult choroid plexus (*Kcna1*, *Kcnj13*) compared with five that were expressed at a higher level in the embryo (*Kcna3*, *Kcna6*, *Kcnj5*, *Kcnj15*, *Kcnk3*). In addition there were seven *Kcn* genes not previously identified in choroid plexus expressed at higher levels in the adult and no less than another twenty-two expressed at a higher level in the embryo (refer to **Table S4**). This high expression of many K^+^ channels is presumably at least in part a reflection of a large requirement for intracellular K^+^ in a rapidly growing brain. Because of difficulties due to changing terminology for Na^+^ and Ca^2+^ channel genes we were unable to confirm the identities of the few such channels that have been shown to be physiologically active in adult choroid plexus (**Table 4**). However, seven Na^+^ channel genes not previously identified in choroid plexus all expressed at a higher level in the embryo. Twelve of thirteen voltage-activated Ca^2+^ genes also not previously identified in choroid plexus were expressed at a higher level in the embryo. Although it is not yet clear that these genes are functionally active, the large number expressed at a higher level in embryonic choroid plexus may reflect the importance of Ca^2+^ for a range of intracellular mechanisms in the developing brain; evidence that at least some of these channels are likely to be active comes from the experiments of Schmitt and colleagues [72] who demonstrated unidirectional calcium transport in the blood-to-CSF direction across choroid plexus epithelial cells *in vitro*. Other channels identified are summarized in **Table 4** included the transient receptor potential cation channels *Trpv4* and *Trpm3,* previously described in adult plexus cells. In addition there were several TRP channel genes not previously described in choroid plexus; these were expressed at a higher level at E15 than in the adult. There were also ligand-gated purinoceptors and volume-activated anion channels (*Clcn4*, *Best3*).

#### Ion transporters and associated enzymes

The data summarised in **Fig. 2** and **Table 5** show that the key contributors to CSF secretion, carbonic anhydrase 2 and three isoforms of Na/K-ATPase (Atp1a1, Atp1a2, *Atp1b1*) known to be present in adult choroid plexus and associated with CSF secretion were already expressed in E15 choroid plexus, albeit at lower levels than in the adult plexus. There were genes for a further two Na/K-ATPases that were both expressed at higher levels in adult choroid plexus than at E15. In addition, genes for several other carbonic anhydrases were identified, including three that were expressed at higher level at E15 and six that was expressed at a higher level in the adult (**Tables 5** and **S2**). Car8 in E15 choroid plexus was expressed at 77-fold above the adult. Although structurally similar to other carbonic anhydrases it has been found to lack enzyme activity [73] and may have other functions: cerebellar movement disorders associated with *Car8* mutations [74] and promotion of colon cancer cell growth [75] have been reported. Of note is that the genes, which were expressed higher or lower than in adult, were expressed at the same level at E19 and at E15 (data not shown). This is in agreement with the developmental increase in CSF secretion rate, which occurs only at or shortly after birth in rat [76], [78].

In adult choroid plexus, gene expression for several of the key transporters shown in previous studies to be involved in CSF secretion were identified (**Fig. 2**, **Tables 5** and **S2**), including *Slc12a2* (*Nkcc1*, Na^+^/K^+^/Cl^−^), *Slc4a5* (*Nbce2*, Na^+^/HCO_3_^−^), *Slc4a10* (*Ncbe*/*NBCn2*, Na^+^/HCO_3_^−^/Cl^−^) and *Slc4a2* (*Ae2*, Cl^−^/HCO_3_^−^). Two transporters previously identified as functional in adult choroid plexus (**Slc4a11**-NaBC1 and Slc4a7-NBCn1, Damkier et al., 2010) were not detected above background levels, but **SLC4A11** has only been detected in human choroid plexus and identification of SLC4A7 depended on the specificity of the antibody used (see also Limitations of study, below). However, other family members were identified (**Fig. 2**, **Table 5**). In E15 choroid plexus, 129 ion transporter genes were identified (**Table S2**). These included 41 *Slc* transporter genes, 31 ATPases and ATP synthase subunit genes, 2 *Abc*, 2 *Slco* and 1 K^+^ transporter gene. Some of these were those known to be functional in adult plexus, but expressed at a lower level at E15 (**Fig. 2**, **Tables 5** and **S2**). However, there were also numerous ion transporter genes not previously identified in choroid plexus, some of which were expressed at a higher level in E15 choroid plexus than in the adult (**Tables 5** and **S2**).

#### Aquaporins

Aquaporin-1 is the best studied of the water channels in choroid plexus epithelial cells. It is located principally in the microvilli of the apical surface of the epithelial cells protruding into the ventricular CSF. It also has a sparse localisation in the baso-lateral membrane [78]. These authors have also shown in several species including human that immunohistochemical staining appears in these characteristic locations as soon as the choroid plexus begins to differentiate. The present results confirm expression of the *Aqp1* gene at least as early as E15 in the rat choroid plexus. There have been several reports of other aquaporins in choroid plexus epithelial cells, but their functional significance is unclear. Here we have identified *Aqp4* present at both ages but at a higher level of expression in adult choroid plexus. *Aqp3* and *Aqp8* were also detected, but only in E15 choroid plexus.

### Immunohistochemistry

In order to establish whether genes identified in the RNA-Seq analysis were translated into their protein products we attempted to examine the cellular distribution of some of their protein products. We chose not to investigate the immunohistochemistry of tight junction genes in this study as this has been reported elsewhere in rat and human development [14]: the expression of CLAUDIN-1, -2 and -3 were shown in the lateral ventricular choroid plexus of embryonic human. In addition CLAUDIN-1 immunoreactivity in the embryonic rat was shown to be similar to that in the adult (in agreement with our expression data). Similarly CLAUDIN-2 immunoreactivity was negligible at E19 in the rat, but was extensive in the adult – as were the levels of *Cldn2* transcript reported above. In addition these authors showed the presence of CLAUDIN-3, -4, -5, -9 and -19 in the developing and adult rat [14].

In the current study we chose instead to examine the cellular distribution of representative antigens from three different categories: ion channels, transporters and associated enzymes. Each of the three genes studied was expressed at a much higher level at E15 than in the adult choroid plexus. Examples of immunohistochemical staining are shown in **Fig. 3**. Carbonic anhydrase-related protein VIII (CA8) shows a strong reactivity localized to extended segments of the epithelial layer of the choroid plexus, which is in contrast to the unstained vasculature and fibroblasts (**Fig. 3A**). Adult choroid plexus lacked CA8 immunoreactivity both in epithelium and endothelium (**Fig. 3B**). Immunoreactivity of SLC4A1, also known as Band 3 Anion Transport Protein, is expressed strongly in both epithelium and vasculature in the entire plexus at E15 including all cell nuclei and cytoplasm (**Fig. 3C**). In the adult plexus SlC4A1 immunoreactivity is mainly nuclear but varying in both epithelium and endothelium (**Fig, 3D**). CLIC3, a chloride intracellular channel 3, is highly expressed in the E15 plexus, localized in the cytoplasm of epithelium and vasculature (**Fig. 3E**). In the adult plexus, blood vessels and connective tissue are devoid of immunoreactivity in contrast to occasional plexus epithelial cells, which show strong cytoplasmic staining (**Fig. 3F**).

The three chosen examples represent an enzyme-like protein of still undefined biological function (CA8), an anion transport protein (SLC4A1) and an ion channel protein (CLIC3) and show very different patterns of immunoreactivity both in the developing and adult choroid plexus. They are all strongly expressed at E15 but their cellular localization varies. In E15 plexus CA8 is confined to the epithelium, SLC4A1 is present in nuclei and cytoplasm of both epithelium and endothelium and CLIC3 is visible in cytoplasm of all choroid plexus cells (**Fig. 3**). There is also a very visible difference between sections of adult plexuses, with no staining at all for CA8, to stained epithelial and endothelial nuclei for SLC4A1 and to cytoplasmic staining of single scattered epithelial cells in case of CLIC3 (**Fig. 3**).

The results obtained from the immunohistochemical analysis also demonstrated that the genes identified in RNA-Seq were mostly localized in choroid plexus epithelium and not in contaminating endothelia derived from plexus stroma. The exception is SLC4A1, which is a well-known marker of erythrocytes; staining obtained in the present study (**Fig. 3C and D**) confirmed this. However it should also be noted that such a possible contamination represents only a small fraction of the total tissue isolated.

### Evidence for function of ion channels, transporters and associated enzymes in developing choroid plexus

Consistent with low expression of Na/K-ATPase and carbonic anhydrase 2 protein in E15 choroid plexus, studies using western blotting and immunohistochemistry have reported levels of both are very low in the E15 rat choroid plexus [79]. Though there is little evidence on the function of Na/K-ATPase and carbonic anhydrase in the developing choroid plexus, it has been shown that activity of Na/K-ATPase in fetal/newborn rabbits is low compared to adults, but was sufficient to establish a gradient for Na^+^ between CSF and plasma [80]. The low expression of *Atp1b1* and other Na/K-ATPases as well as *Car2* in E15 rat choroid plexus is consistent with a low CSF secretion rate in rat [76], [77] and other species [81] early in brain development. The higher expression of several carbonic anhydrases, particularly *Car8* (77-fold) in E15 choroid plexus compared to the adult is surprising; if functional, it may be important for some biological activity other than the low level of CSF secretion, related for example to the higher level of CO_2_ in the fetus [81]. According to Picaud and colleagues however, it lacks the characteristic activity that would be predicted from its structure [73].

Data in **Table 6** show the distribution of ions between CSF and plasma in both the young and adults of a range of species: rat (newborn [82], adult [82], [83]) ; monkeys [63]; rabbits [82]; and sheep [64]. The choroid plexus epithelium mediates net secretion of Na^+^, Cl^−^ and HCO_3_^−^ ions, as well as H_2_O into the CSF. The presence of ionic gradients between CSF and blood plasma indicates that intercellular tight junctions are functionally effective and the presence of an effective pump for the ions for which there are gradients. These data show that ion gradients between CSF and plasma are established early in brain development, but they change during development, presumably in relation to specific features of brain development. Though there is little information on the movement of Ca^2+^ and Mg^2+^ ions across the choroid plexus, many transient receptor potential vanilloid (TRPV) channels were detected (see **Table S1** and **S2**). These channels are known to be selective for Ca^2+^ and Mg^2+^ over Na^+^ ions. We report the presence of *Trpv* transcripts at E15 and in the adult will little change in expression (e.g. *Trpv1, Trpv3*) – as reported immunohistochemically in the developing rat by Jo and colleagues [84]. *Trpv4* was highly expressed (raw count 3020) and enriched over 9-fold in the adult (**Table 4** and **S2**), in contrast, *Trpv2* and *Trvp6* were enriched in the embryo, but expression was also comparatively low (raw counts below 100, see **Table S1**).

**Table 6:** Electrolyte concentrations in CSF and plasma of a range of species.

Species	Age	Ions (CSF/Plasma)					
		Na^+^	K^+^	Cl^−^	HCO_3_^−^	Ca^2+^	Mg^2+^
Rat (21d)[82]	NB	144/150	3.9/6.5	108/102	24.3/25.5 [114]	2.4/2.9 [115]	-
	Adult	152/153	3.2/5.0	125/105	25.8/20.5 [116]	1.4/2.5 [115]	-
Monkey (168d) [63]	E50-60	-	3.6/4.5	-	-	3.8/4.6	1.9/1.2
	Adult	-	2.6/4.5	-	-	2.3/4.6	1.9/1.2
Rabbit (28d) [82]	P20	143/143	4.1/3.1	113/102	-	-	1.5/3.2
	Adult	152/155	3.0/3.2	120/102	-	-	1.5/2.2
Sheep (150d) [64]	E44-50	135/138	5.4/-	113/113	-	-	2.0/1.6
	E85-90	144/136	3.5/3.8	123/104	-	3.4/6.1	1.9/1.8
	Adult	148/138	3.1/3.4	128/115	-	2.5/3.4	1.8/1.4
Dog (62d) [112]	P1-4	149/141	3.3/4.3	122/104	26.5/25.6	-	-
Horse (340d) [113]	P1-2	143/-	3.6/-	109/-	-	-	-

Electrolyte concentrations (mequiv/L or kg H_2_0) in CSF and plasma of embryos/newborns and adults. Gestation term for each species listed in days in brackets. Note that there is net movements of sodium, chloride and bicarbonate ions into the CSF, and a net movement of potassium ions into the blood. Net water movement into the CSF is by aquaporin channels.

The presence of an ion gradient indicates that the intercellular pathway between adjacent choroid plexus epithelial cells is closed to significant ion transfer (i.e. the tight junctions are functionally effective) and the relevant ion pumps and channels are active. Thus there is physiological evidence that ion pumps are active early in brain development, but the function of individual ion transporters and channels in developing choroid plexus remains to be investigated as has been done in adult plexus [67], [72]. Preliminary studies have shown that this is technically feasible at least as early as newborn rat using standard patch clamping methods (NRS, unpublished).

### Genes related to synaptic structure and function

A surprising finding in the RNA-Seq dataset is a large number of genes related to synaptic structure and function (**Table S5**). For reasons discussed below they are very likely to be expressed solely in choroid plexus epithelia cells rather than due to contamination from the very small amount of non-epithelial tissue in the whole choroid plexus used for this study. At E15 there were 64 genes defined by their GO categories as synapse-related and expressed at a higher level than in adult plexus (**Table S5**). Of these, more than half (28) were genes for neurotransmitter receptors, particularly glutamatergic (14) and GABA-ergic (5) but also cholinergic (4), serotonergic (2) purinergic (2) and adrenergic (1). In contrast in the adult choroid plexus only one glutamate, two GABA and two purinergic receptors were expressed at a higher level than at E15. At both ages there was a large number of genes whose proteins are known to be associated with synaptic structures were expressed at a higher level in the E15 choroid plexus (e.g. *Syn1, Syn3, Nrgn, Stx1z*a, *Synpo*), and synaptic vesicle transcripts were expressed at a higher level in adult plexus (e.g. *Sv2b, Syngr1, Snap25, Vamp1, Syp Syt1, Syt4, Snapin*).

It remains to be shown how many of these genes are functionally active in both the developing and adult choroid plexus, but these findings raise the unexpected possibility that functional (secretory) activity of choroid plexus epithelial cells may be controlled by a complex set of neurotransmitters.

### Barrier permeability

Many still believe that brain barriers in the embryo and newborn are immature and (by implication) dysfunctional [85]–[87] (for review see [6]). One reason is that early in brain development the concentration of proteins in CSF is high and these proteins are mostly derived from blood plasma [5], [6]. This has been interpreted as reflecting a passive “leak” across incomplete brain barrier interfaces [88]. An alternative explanation is based on measurements of CSF volume of distribution [9], [17] and evidence of active transfer of proteins from blood into CSF across choroid plexus epithelial cells by an intracellular pathway [10], [17], [89]–[93].

**Figure 4:**
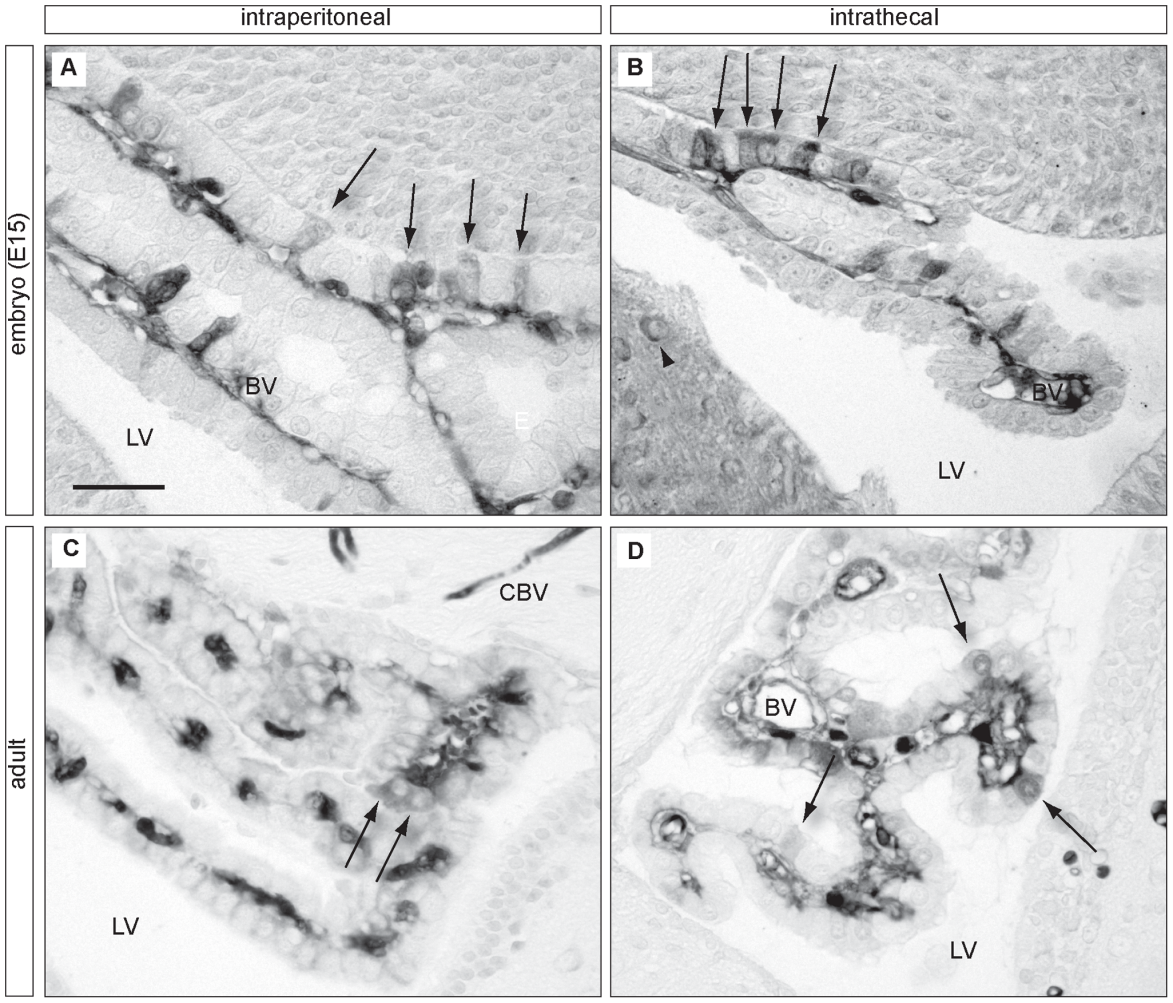
Localisation of biotinylated dextran amine (3000Da) in choroid plexus epithelial cells. Tight junctions between choroid plexus epithelial cells are functionally mature from very early in development as seen by exclusion of a 3000Da biotinylated marker following intraperionteal (**A**, **C)** or intrathecal (**B**, **D**) injection. **A** and **C,** a subset of plexus epithelial cells was seen to take to marker up from the blood and transport it to the CSF (arrows). In the adult the marker was also present in the cerebral vasculature (CBV). **B** and **D**, were following injection into a lateral ventricle; many more plexus epithelial cells (approximately double the number) were seen to take up the marker from the CSF and transport it into the blood stream. Some neurons in the ventricular zone were also seen to take up the marker from the CSF (arrowhead in **B**). Scale bar: 25 µm for all. Abbreviations: BV, blood vessels; CBV, cerebral blood vessel; CSF, cerebrospinal fluid; LV, lateral ventricle.

**Figure 5:**
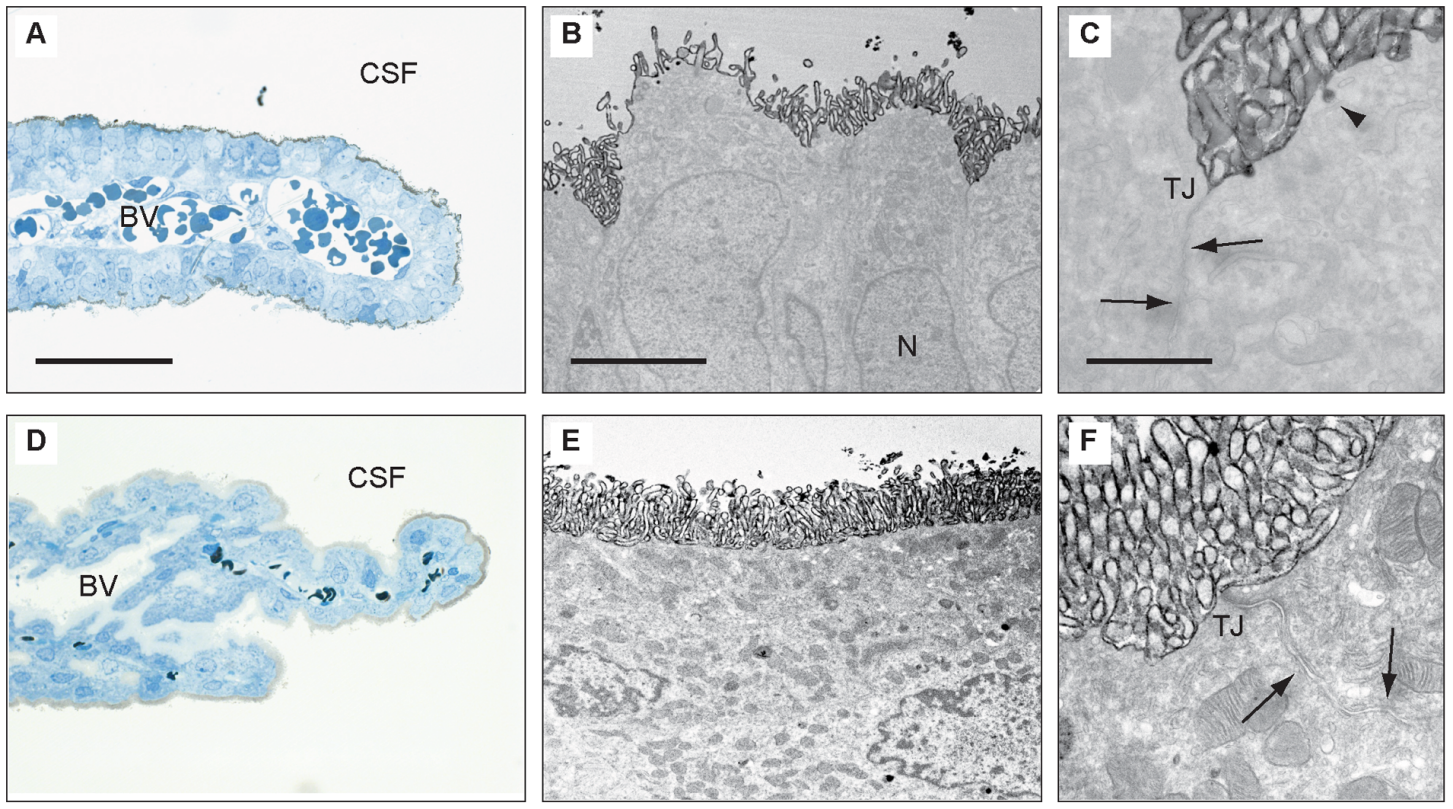
Localisation of biotin ethylenediamine (BED, 286Da) following intraventricular injection. The small tracer, biotin ethylenediamine, was injected intraventricularly to assess barrier function in choroid plexus in the E15 rat embryo (**A–C**) and adult (**D–F**). **A** and **D** are semithin sections stained with toluidine blue; **B**, **C**, **E** and **F** are electron micrographs. Note that the tracer was abundant on the outside of epithelial cells but was restricted by the epithelial tight junctions (TJ), present towards the apical side of these cells and was not visible further into the intercellular cleft. These data suggest that the tight junctions present between intimately apposed plexus epithelium are functional in E15 animals to the same extent as in the adult. Scale bars: A, D = 50 µm, B, E = 5 µm, C, F = 1 µm. Abbreviations: BV, blood vessel; CSF, cerebrospinal fluid; N, nucleus; TJ, tight junction.

One of the main problems with many studies dealing with brain barrier properties is the lack of a comprehensive approach that combines molecular (biochemical) and physiological techniques in the same animal model. To consolidate the data provided by the transcriptome of the blood-CSF barrier, we completed permeability experiments to illustrate the route of entry for water-soluble molecules of a range of molecular weights (from 286 Da and 3 kDa). For experiments at the light microscopic level, embryonic and adult rats were injected with biotinylated dextran amines of 3 kDa either into the blood space (intraperitoneal) or into the CSF (intrathecally). Microscopical localisation of the biotin marker showed that at both ages and following either route of administration, the reaction product was visible in individual choroid plexus epithelial cells and not between the plexus cells (arrows in **Fig. 4**) confirming previously published data for a marsupial species that these cells can transfer water-soluble molecules intracellularly, but there is no leakage between adjacent cells [17], [47]. Samples of CSF and plasma were also collected to confirm the presence of the marker in both compartments indicating transfer of the biotinylated marker across the blood-CSF barrier. Following intraperitoneal injection, CSF samples showed biotin reaction product (not shown), while plasma samples taken from animals that received an intrathecal injection showed a positive reaction for biotin (visible in tissue sections in **Fig. 4B and D**). These data suggest that the 3 kDa water soluble marker is transferred through choroid plexus epithelial cells in both a blood-to-CSF and in a CSF-to-blood direction. To ensure no leakage was occurring between cells, functional integrity of the choroid plexus barrier was visualised at the ultrastructural level with a low molecular weight tracer (biotin ethylenediamine, 286 Da) injected directly into the ventricles of E15 and adult rats and plexus tissue was examined by electron microscopy. As seen in **Fig. 5**, tracer was extensively visible within the microvilli of the epithelial cells but not visible in the intercellular space between epithelial cells. The marker did not penetrate between adjacent epithelial cells as it was halted by the presence of functional tight junctions (arrows in **Fig. 5**). In both the embryonic and adult choroid plexus the tracer was only present on the CSF side of the epithelial tight junctions. Small vesicles containing the tracer were common close to the apical cell membrane (arrowhead in **Fig. 5C**). These data suggest that small water-soluble markers are not able to pass the tight junctions between adjacent plexus epithelial cells in the embryo and in the adult, indicating that they are functionally mature from very early in brain development.

## Limitations of Study

The current study provides an exhaustive analysis at the transcriptome of the rat lateral ventricular choroid plexus of the embryonic and adult rat. The dataset makes available a wealth of information to be mined by researchers from a range of backgrounds including developmental biology, barrier biology and drug delivery. There are three limitations that need to be acknowledged regarding the present study. The first limitation concerns the use of whole choroid plexus tissue. However, the epithelium is the predominant cell type, representing up to 90% of the plexus tissue [13], [18]. The absence of transcript for *Cldn5* (a brain barrier cerebral endothelial-specific claudin family member) in the current dataset suggests that any possible contamination is below detectable limits. In addition, the immunohistochemical evidence for presence of an ion transporter (SLC4A1) an ion channel (CLIC3) and a carbonic anhydrase (CA8) that were found to be distributed predominantly if not exclusively in epithelial cells but not the endothelium or stroma (**Fig. 3**) reinforces this conclusion. A second limitation concerns the bioinformatics analysis of the RNA sequencing database. The analysis of large RNA sequencing databases is quite new and relies heavily on the accuracy of the annotations for each species being studied. Although the rat genome is well annotated, and the number of unknown genes is extremely low, there is still the possibility that some transcripts are missed. In this study only a very limited number of false negative genes were identified, e.g. *Cldn3*. Nevertheless, this is a minor concern that can be rectified with future re-analysis if required when new annotations become available – outlining one of the strengths of high throughput RNA sequencing. A third limitation is that expression data alone do not necessarily equate to functional activity. The immunohistochemical evidence in this paper and in earlier reports [14] shows that at least some of the mRNAs are being translated to protein. The summary of evidence on ion gradients between CSF and plasma provides important evidence that the genes underlying key CSF secretory mechanisms are indeed functional as early as E15 in rat embryos.

In order to provide some direct functional understanding of this complex expression data set we have carried out some permeability experiments using markers that can be visualised down to an ultrastructural level. This confirms earlier work showing that tight junctional impermeability to small molecules is established very early in choroid plexus development. Otherwise we have had to rely on the wealth of published data on junctions associated genes and their proteins to provide a detailed appraisal of the significance of the expression data. One drawback is that much of the literature is based on cell culture studies, without much evidence of the extent to which findings apply to epithelia in general or choroid plexus in particular, *in vivo* (see for example the recent series of articles edited by Fromm & Schulzke [94]).

## Significance

It is a central dogma of epithelial biology that the paracellular pathway through tight junctions is permeable to small water-soluble molecules [95] but the extent may be variable [96]. This view originated from the ingenious experiments of Frömter & Diamond [97] using extracellular microelectrodes to localise low resistance pathways across epithelia at or close to intercellular junctions from which they proposed a paracellular pathway for ions and water. This was a paradigm shift in understanding of the mechanism of transepithelial permeability, which had previously been thought to be transcellular for ions and water. These authors also suggested that small water-soluble molecules might also cross epithelia via the paracellular pathway [97], but later work by Diamond acknowledged an alternative explanation of a small population of low resistance cells accounting for the low resistance pathway [98]. An overall problem with the concept of the tight junction and associated components of the paracellular pathway as a permeability route for small water-soluble molecules across an epithelial interface is that for many years there have been only a few small electron dense extracellular markers available to visualize this pathway; these often showed that this pathway was closed to these molecules [99]. However, as they are usually only applied for very short times because of their toxicity (lanthanum or pyroantimonate), for example by local vascular or parenchymal injection or even in fixed materials it is unclear whether the results apply *in vivo*.

On the other hand, markers commonly used for physiological permeability studies in epithelia (e.g. ^14^C-sucrose) have not been visualised at the electron microscopical level. Our previous ultrastructural results from studies of neonatal marsupial opossums showed that biotin-labelled small molecular sized surrogates for sucrose and inulin are seen in a small proportion of choroid plexus epithelial cells, whether applied on the blood [47] or the CSF [9] side of the plexus, and do not appear to permeate tight junctions. This has now been confirmed for embryonic and adult rat choroid plexus in the experiments reported here (**Fig. 4** and **5**).

The present results from the choroid plexus transcriptome provide a comprehensive identification of junctional protein gene expression. In combination with current and previous studies of permeability in embryos and in adults [5], [9], [47], [91] and supported by results in postnatal [33] and embryonic mice [13], these data strongly indicate that the brain develops within a well-protected internal environment and the exchange between the brain and the periphery is not through incomplete barriers. The early expression of many transporter and channel genes known to be involved in CSF secretion, coupled with published data on ionic gradients between CSF and plasma also shows that a characteristic internal environment is established early in brain development.

Detailed electrophysiological studies in embryonic choroid plexus cells will be required to confirm which transporters and channels are involved in the establishment and control of this internal environment in the embryonic brain. Such studies will be needed to define the essential characteristics of exchange mechanisms in brain barrier interfaces that determine properties of the internal environment of the brain. It remains to be shown how specific properties of this internal environment contribute to brain development and whether deficiencies in these mechanisms may contribute to maldevelopment of the brain or neurological disorders later in life.

## Supporting Information

Table S1 **Complete Excel spread sheet.** The Excel spread sheet containing the comprehensive raw transcript count dataset from Illumina platform. Values refer to individual counts for each transcript. ‘AVE’ columns are average data for each age.
(XLSX)

Table S2 **Full normalised dataset.** A dataset containing all normalised data with differential expression between embryo (E15) and adult.
(XLSX)

Table S3 **Junction specific genes.** A dataset containing junction transcripts from Illumina screen that were statistically enriched in either the embryo (E15) or adult, as identified by GEO annotations.
(XLSX)

Table S4 **Transporters and channel specific genes.** A dataset containing transport and channel specific transcripts from Illumina screen that were statistically enriched in either the embryo (E15) or adult, as identified by GEO annotations.
(XLSX)

Table S5 **Synaptic transcripts.** A dataset containing synaptic transcripts from Illumina screen that were statistically enriched in either the embryo (E15) or adult, as identified by GEO annotations.
(XLSX)
