# Correction: Mechanisms That Determine the Internal Environment of the Developing Brain: A Transcriptomic, Functional and Ultrastructural Approach

**DOI:** 10.1371/journal.pone.0147680

**Published:** 2016-01-19

**Authors:** Shane A. Liddelow, Katarzyna M. Dziegielewska, C. Joakim Ek, Mark D. Habgood, Hannelore Bauer, Hans-Christian Bauer, Helen Lindsay, Matthew J. Wakefield, Nathalie Strazielle, Ingrid Kratzer, Kjeld Møllgård, Jean-François Ghersi-Egea, Norman R. Saunders

The published article contains errors in Fig 2 and its caption. Please see the correct Fig 2 and its caption here.

**Fig 2:**
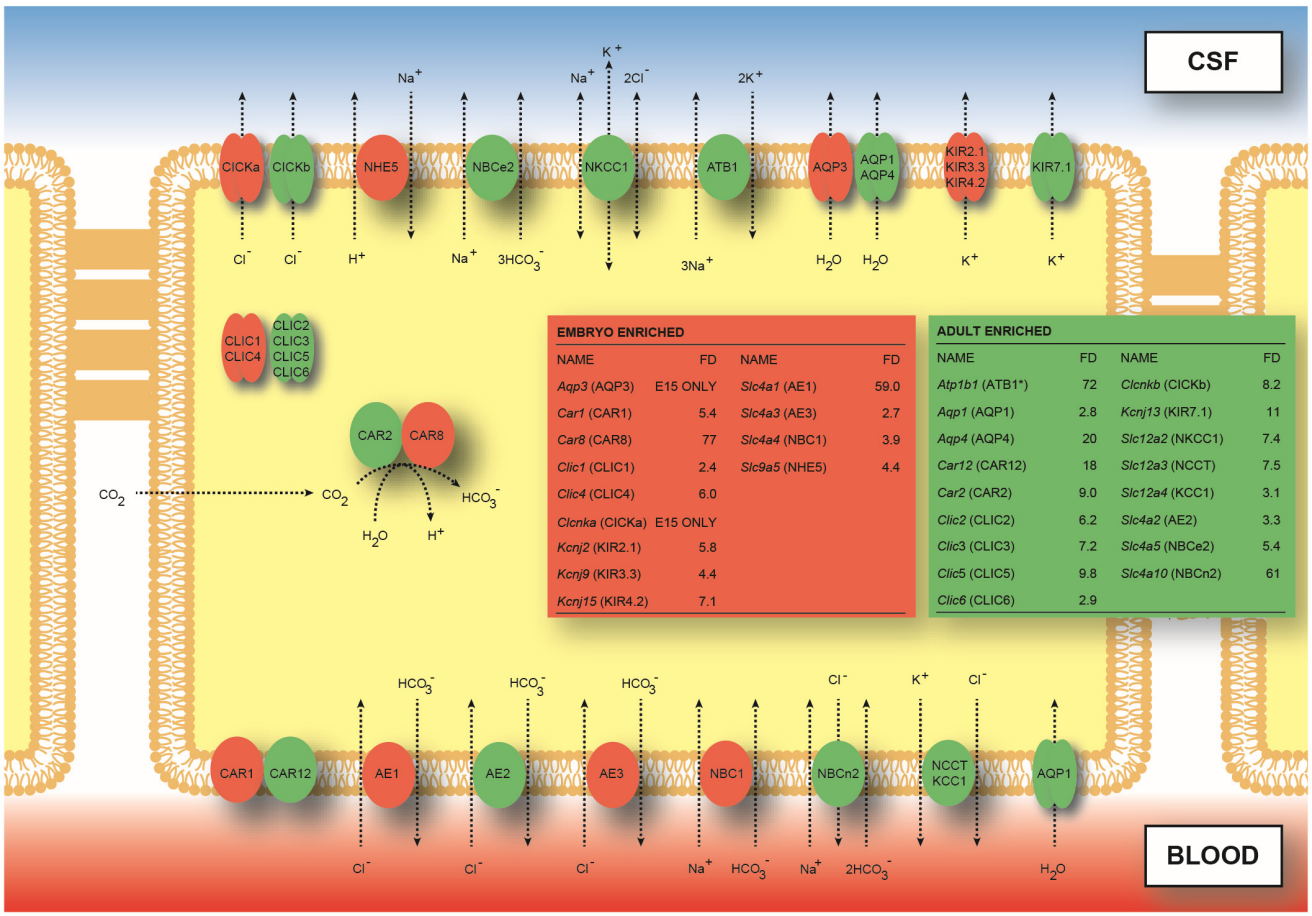
Localization of proteins for ion transporters, channels and associated enzymes and identification of their corresponding genes in adult and immature rat choroid plexus. Data for the localisation of the proteins are from Damkier et al. (2010) and see also Tables 3 and 4. CSF secretion results from coordinated transport of ions and water from basolateral membrane to cytoplasm, then sequentially across apical membrane into ventricles (for review see Davson & Segal, 1996). On the plasma-facing membrane is parallel Cl^-^/HCO_3_^-^ exchange (AE2, *Slc4a2*) and Na^+^/HCO_3_^-^ co-transport (NBC1, *Slc4a4*) with net function bringing Cl^-^ into cells in exchange for HCO_3_^-^ (Murphy & Johanson, 1989). Also basolaterally located is Na-dependent Cl^-^/HCO_3_^-^ exchange (NCBn2 *Slc4a10*) that modulates pH and perhaps CSF formation (Damkier et al., 2007). Apical Na^+^ influx by NHE5 (Slc9a5) and ATB1 (*Atb1b1*, Na^+^/K^+^-ATPase, asterisk) maintains a low cell Na^+^ that sets up a favorable basolateral gradient to drive Na^+^ uptake (Pollay et al., 1985). Na^+^ is extruded into CSF mainly via the Na^+^/K^+^-ATPase pump (ATB1, *Atb1b1*) and, under some conditions, the Na^+^/K^+/^-Cl^-^ co-transporter NKCC1, *Slc12a2*, see Johanson et al., 2008 for review). Overall cell volume is maintained by the K^+^/Cl^-^ co-transporters NCCT (*Slc12a3*) and KCC1 (*Slc12a4*). Aquaporin (AQP1/3/4) channels on CSF-facing membrane mediate water flux into ventricles (Oshio et al., 2005). Polarized distribution of carbonic anhydrase (CAR) and Na^+^/K^+^-ATPase, and aquaporins, enable net ion and water translocation to CSF (see Johanson et al., 2008 and Brian et al., 2010 for review). The gene *Slc4a7* (NBCn1) was not detected by RNA-Seq, although it has been reported in both rat and mouse choroid plexus (Praetorius et al. 2004); this may have been for technical reasons or because of lack of antibody specificity see section “Limitations of study”. The genes for *Clir* (chloride inwardly rectifying) channels has not been previously identified but are probably *Clica* and *Clicb*. The gene for VRAC (volume regulated anion channels) is not known (Alexander et al., 2011); see also Table 4. The carbonic anhydrases CAR2 and CAR8 have an intracellular distribution; CAR8 has been shown to lack the characteristic enzyme activity of these proteins (Picaud et al., 2009). It is not known whether it is functional in the embryo. The CLIC chloride channels are also intracellular (Edwards & Kahl, 2010) and as such we have placed them cytosolically, however it is more likely that they sit on the internal membrane of the cell and aid in movement of Cl^-^ to other channels. The inset boxes show the fold differences for genes expressed at a higher level in the embryonic (red) or in the adult (green) choroid plexus.
